# Activation of CD44 signaling in leader cells induced by tumor-associated macrophages drives collective detachment in luminal breast carcinomas

**DOI:** 10.1038/s41419-022-04986-4

**Published:** 2022-06-09

**Authors:** Feng Gao, Guoliang Zhang, Yiwen Liu, Yiqing He, Yumeng Sheng, Xiaodan Sun, Yan Du, Cuixia Yang

**Affiliations:** 1 Department of Clinical Laboratory, Shanghai Jiao Tong University Affiliated Sixth People’s Hospital, Shanghai, 200233 China; 2 Department of Molecular Biology Laboratory, Shanghai Jiao Tong University Affiliated Sixth People’s Hospital, Shanghai, 200233 China

**Keywords:** Breast cancer, Cancer microenvironment

## Abstract

Collective detachment of cancer cells at the invading front could generate efficient metastatic spread. However, how cancer cell clusters shed from the leading front remains unknown. We previously reported that the dynamic expression of CD44 in breast cancers (BrCas) at collectively invading edges was associated with tumor-associated macrophages (TAMs). In this study, we first observed that the highly expressed CD44 (CD44^high^) cancer cell clusters were located in the BrCa circulating vessels, accompanied by CD206^+^ TAMs. Next, we identified that the cancer cell clusters can be converted to an invasive CD44^high^ state which was induced by TAMs, thus giving rise to CD44-associated signaling mediated cohesive detachment. Then, we showed that disrupting CD44-signaling inhibited the TAMs triggered cohesive detaching using 3D organotypic culture and mouse models. Furthermore, our mechanistic study showed that the acquisition of CD44^high^ state was mediated by the MDM2/p53 pathway activation which was induced by CCL8 released from TAMs. Blocking of CCL8 could inhibit the signaling cascade which decreased the CD44-mediated cohesive detachment and spread. Our findings uncover a novel mechanism underlying collective metastasis in BrCas that may be helpful to seek for potential targets.

## Introduction

Tumor cells can invade and disseminate in vivo in collective way, which is more efficient in developing metastases than moving as single cells [1, 2]. The frequency of collective cancer cell clusters in the invading front has been linked to metastatic potential and poorer prognosis in breast cancer, which might be the origin of circulating cancer cell clusters (CTC clusters) [3–5]. Shift of cohesive invasion/collective detachment at the invading edge within tumors induces a switch toward collective metastasis and polyclonal seeding [6, 7]. Of which, the acquisition of the capacity to escape from primary tumor sites in a cohesive multicellular way is the most important stage in initiating collective detachment. However, how the transition from collective invasion to cohesive detachment remains unclear.

CD44, as a mesenchymal marker and a multifunctional cell surface glycoprotein, plays a critical role in tumor progression and specifically in collective invasion in luminal-like BrCa [8–10]. CD44 is a putative marker of breast cancer stem cells (CSCs) [11], and changes of its expression are implied in the transit between non-stem cancer cells (non-CSCs) and CSCs or shift between epithelial-CSCs and mesenchymal-CSCs [12–14], conferring to the high flexibility of cancer cells. Mesenchymal CD44 expression also contributes to the acquisition of partial EMT phenotypes with more invasive capability, displaying an inducible feature of CD44 in regulating cell plasticity. Our previous study has shown that, at the onset of collective cell invasion, leader cells featured by a CD44^high^ state arise from the CD44^low^ subsets at the inner part of tumor bulk [8]. The dynamic switching of CD44 triggers the transition of less aggressive luminal BrCas to invasive state. Moreover, a recent study reported that the intercellular CD44-CD44 homophilic interactions could mediate multicellular aggregation in circulation [15], further suggesting the contribution of CD44 in maintaining multicellular dissemination. However, the role of the dynamic shift of CD44 in cancer cells collective detachment from primary tumor mass to circulation is poorly understood.

While many molecular cues from tumor microenvironment (TME) controlling single-cell dissemination have been described [16, 17], little is known about the interactions between invading cell clusters and TME. Indeed, while the cell clusters in circulation, also known as “tumor microemboli”, are thought to come from cohesive dissemination from the primary tumor site, however, what causes the BrCa cell clusters to collectively detach is obscure. Tumor-associated macrophages (TAMs) potently promote metastasis of BrCa [18–20], with a high heterogeneity depending on particular subtype of BrCa [21]. However, whether and how TAMs trigger the collective detachment of cancer cells from invading edge is still largely unknown. Meanwhile, we previously reported that M2-type TAMs which can promote tumorigenesis by secreting premetastatic cytokines, appeared in peripheral blood [22] and invading edges [23]. Intriguingly, we also found that TAMs could induce CD44 switching from the CD44^low^ to CD44^high^ state [8]. However, the CD44^high^ state of leader cells induced by TAMs and its effects on cancer collective detachment and metastasis are less investigated.

In the present study, we used in vitro and in vivo models to identify the role of CD44^high^ state in leading cell clusters and its associated signaling induced by TAMs in collective detachment. Using 3D organotypic culture and mouse models, we showed that disruption of CD44-signaling decreased the cohesive detachment induced by TAMs-derived CCL8. Importantly, mechanistic studies revealed that the CD44-dependent multicellular dissemination is initiated by an MDM2-p53 signaling cascade. These data suggest a new finding explaining the plasticity of invading cell clusters in luminal breast carcinomas.

## Results

### The co-location of CD44^high^ cancer cells and TAMs in microemboli in vessels and at invading edges of human BrCas tissues

**Fig. 1:**
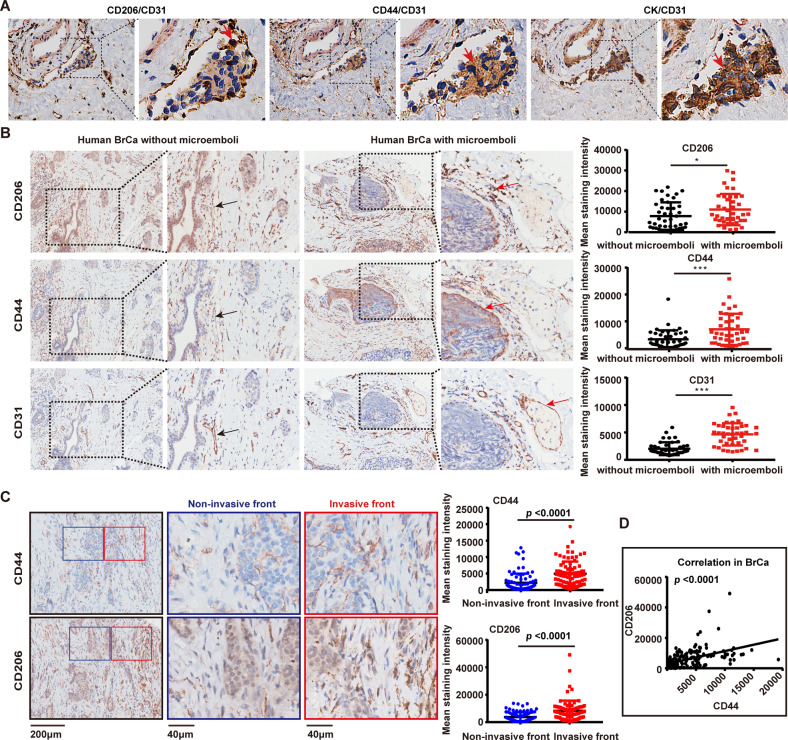
CD44^high^ cancer cells and TAMs co-localized in microemboli of vessels and at invading edges of human BrCa tissues. **A** Representative images of cell clusters in the vasculature of human primary breast tumor. The distributions of CD206 (brown), CD44 (brown), CK (brown), and CD31 (red, indicating vessels) in tissue serial sections from luminal-like BrCa (*n* = 15) were determined by immunohistochemical staining. Nuclei are stained with hematoxylin. **B** Immunohistochemical staining of CD206, CD44, and CD31 was performed in serial tissue sections of human BrCa. Tumors with and without microemboli from a tissue microarray (85 human breast lesions) were analyzed. Using Image-Pro Plus software, the expression intensity of each molecule was evaluated by integral optical density and compared with and without microemboli. Data were analyzed with the Mann-Whitney test. *p < 0.05, ***p < 0.001. **C** The expression of CD44 and CD206 in the invasion front versus non-invasive front of serial sections from human BrCa tissues was analyzed. Data represents the mean ± SEM. Mann-Whitney test. **D** A positive association between high frequency of CD206^+^-macrophage and CD44^high^-cancer cells in BrCa.

To detect the expression of CD44 and the marker of TAMs in microemboli in vessels in human BrCas tissues, CK, CD206, CD44, and CD31 were used for immunohistochemical staining. The results showed that clustered cancer cells were detected within the vasculature of tissue sections from human luminal-like BrCa. CD44^high^ cancer cells and CD206^+^ macrophages were observed in the clusters simultaneously (Fig. 1A). Additionally, to analyze the expression of CD44 or CD206 between tumors with and without microemboli groups, IHC staining was performed on a tissue microarray (85 human breast lesions). Results showed that the expression of CD206, CD44, and CD31 in the BrCas tissues with microemboli was significantly higher than that in BrCas without microemboli (Fig. 1B and Table S1), suggesting that CD44^high^ cancer cells and CD206^+^ macrophages were associated with the occurrence of cohesive detached cell clusters in the circulatory system.

To further analyze the relationship between CD44^high^-cancer cells and CD206^+^ TAMs, matched tumor inner and invasive fronts were analyzed in luminal-like BrCas sections with or without microemboli. Intriguingly, we found that both CD44 and CD206 were mainly expressed at the tumor invasive front, with significantly weak expression in the tumor inner (Fig. 1C). More importantly, a positive correlation between CD44 and CD206 expression was observed, implying an intimate association of high frequency between CD44^high^ cancer cells and CD206^+^ macrophages at the invasive edges (Fig. 1D).

### The acquisition of CD44^high^ state in collective detachment

**Fig. 2:**
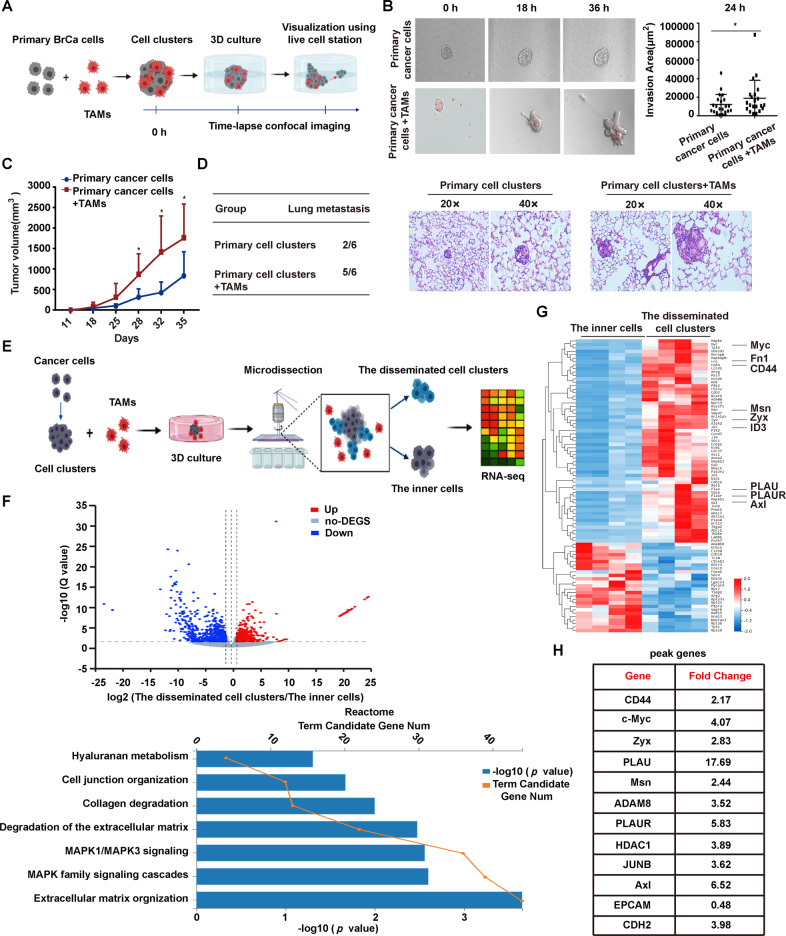
The acquisition of CD44^high^ state in collective detachment upon TAMs stimulation. **A** Illustration of the 3D cohesive disseminating assay. Primary BrCa cells (EpCAM^+^/CD45^−^) and TAMs (EpCAM^−^/CD45^+^/F4/80^+^/CD206^+^) purified from MMTV-PyMT tumors by FACS were cultured in ultra-low attachment plates to spontaneously aggregate into multicellular clusters. Then cell clusters were cultured in 3D collagen matrix (embedded into Matrigel/Collagen-I) with or without TAMs. The collective detachment was observed directly by time-lapse confocal microscopy. **B** Cohesive dissemination of primary BrCa cell clusters after co-cultured with TAMs for 36 h. Images represent 3 independent experiments. TAMs were prestained with Vybrant CM-DiI (red). **C** Tumorigenicity of multicellular clusters from MMTV-PyMT tumors with or without TAMs following orthotopic injection into SCID mice (*n* = 6/group). Tumor growth was monitored and recorded. Data are represented as the mean ± SEM. *p < 0.05 for clusters plus TAMs group compared with cell clusters only group, *t*-test. **D** Five weeks after injection, the number of mice that developed subcutaneous tumors and lung metastasis in each group was counted. Lung metastasis was determined by HE. More lung metastasis was observed in cell clusters plus TAMs group than that in the control group. **E** Schematic representation of laser microdissection (Leica LMD6 system) to obtain collective disseminated leader cells and the inner cells separately. RNA sequencing analysis (Illumina HiSeq X-Ten) was conducted to detect the gene expression in the disseminated cell clusters for comparison with gene expression in the inner cells of cell clusters. TAMs were pre-stained with Vybrant CM-DiI (red). **F** Volcano plot of the RNA-sequencing differential gene expression between the disseminated cell clusters and the inner cells from multicellular clusters using DE-seq2, which was co-cultured with TAMs in top-3D system with log two-fold change >1 or <−1, and *p* < 0.05. The red dots are the genes that were significantly upregulated in disseminated cells, and blue dots are the genes downregulated. Reactome functional annotation shows the enriched GO terms for the genes upregulated or downregulated upon TAMs-induced cohesive dissemination. **G** Heat map of unsupervised hierarchical clustering of 520 invasion-related genes are shown to display differentially expressed genes in the collective disseminated cell clusters and the inner cells purified from TAMs-stimulated primary BrCa cell clusters (red and white indicate higher and lower expression, respectively) with fold changes ≥2 and FDR ≤ 0.05. **H** The differentially upregulated transcripts in disseminated cells from RNA-sequencing analysis were shown.

To investigate the alteration of CD44 in cohesive detachment of BrCas, an aggressive mouse breast cancer model (MMTV-PyMT) most commonly used for collective invasion and metastasis study was applied [6, 8, 24]. Primary breast cancer cells (EpCAM^+^/CD45^−^) and TAMs (EpCAM^−^/CD45^+^/F4/80^+^/CD206^+^) were isolated and cultured in suspension separately for 48 h modified from a previous report [1]. Then equal numbers of cancer cell clusters were embedded with or without Vybrant CM-DiI (red) labeled-TAMs in 3D or top-3D Collagen/Matrigel-rich gels to observe the cohesive detachment of cell clusters, which was monitored by time-lapse microscopy as schematically represented in Fig. 2A. Results showed that, 2 days later, clusters co-cultured with TAMs were detached with more spreading area and invasive fingers in 3D or top-3D culture system (Fig. 2B, Video S1 and 2), indicating that TAMs promoted the cell detachment as cohesive multicellular clusters. Further, equal numbers of cancer cell clusters with or without TAMs were injected orthotopically into the mammary fat pad of SCID mice. The cancer cell clusters mixed with TAMs displayed a relatively stronger tumor-initiation capability (Fig. 2C) and generated more lung metastases than cell clusters only (Fig. 2D), indicating that TAMs significantly enhanced the metastatic spreading of multicellular clusters in vivo.

Consistent with these in vivo observations, TAMs-stimulated multicellular clusters had a higher spreading area than naïve clusters in top-3D basement membrane-rich gels (Fig. S1). After being stimulated by TAMs for 24 h, the disseminated leader cells and the inner cells of multicellular clusters cultured in top-3D basement membrane-rich gels were collected by laser capture microdissection (LCM) separately (Fig. 2E). Then, RNA sequencing was performed to analyze gene signatures of detached cell clusters. 1753 genes were shown to be differentially expressed between the two clusters (R2-fold; *p* < 0.05) (Fig. 2F). The differentially expressed genes were presented by unsupervised hierarchical clustering assay including focal adhesion, cell junction, and MAPK signaling pathway (Figs. 2F and S2A). Gene Ontology (GO) and Kyoto Encyclopedia of Genes and Genomes (KEGG) functional enrichment analysis showed that distinct cascades were enriched in the disseminating leader cells (Figs. 2G and S2B). Consistent with the high invasiveness of disseminated cells, several genes related to partial EMT, invasiveness, and maintenance of cell-cell contact were found to be expressed significantly among the most differentially expressing ones including CD44, Zyx, Axl, Msn, ID3, Fn1, PLAU, PLAUR, and Myc, which were conversely expressed at lower levels in the inner cells of clusters than in disseminated leader cells (Fig. 2G). These data showed that TAMs could stimulate breast cancer cell clusters by rewiring their transcriptome to alter invasive gene expression, resulting in collective detachment. Further analysis of TAM-stimulated primary cancer cell clusters compared to naïve cell clusters revealed that c-Myc, PLAU, PLAUR, and AXL (Figs. 2H and S2C) were significantly upregulated, while the downregulated epithelial marker (EPCAM) was observed in the disseminated cell clusters. As previously reported, PLAU and its receptor PLAUR were involved in regulating cell adhesion and promoting tumor cell migration [25, 26], while c-Myc and AXL were associated with EMT, invasiveness, and migration [27, 28]. Notably, CD44 was also upregulated in the disseminating leader cells and was reported to be associated with the functions of these genes (c-Myc, PLAU, PLAUR, and AXL) [29, 30]. Moreover, from our previous study, CD44 switching was found to be related to the stemness and plasticity of cancer cells, showing the capability to give rise to collective invasion [8]. Therefore, we hypothesized that the acquisition of CD44^high^ state might contribute to the cohesive dissemination of cell clusters, in which some tumor invasive-related genes may also be involved that needs our further confirmation.

### The essential role of CD44^high^ state and CD44-related cell-cell junction molecules in cohesive detachment of BrCa cells

**Fig. 3:**
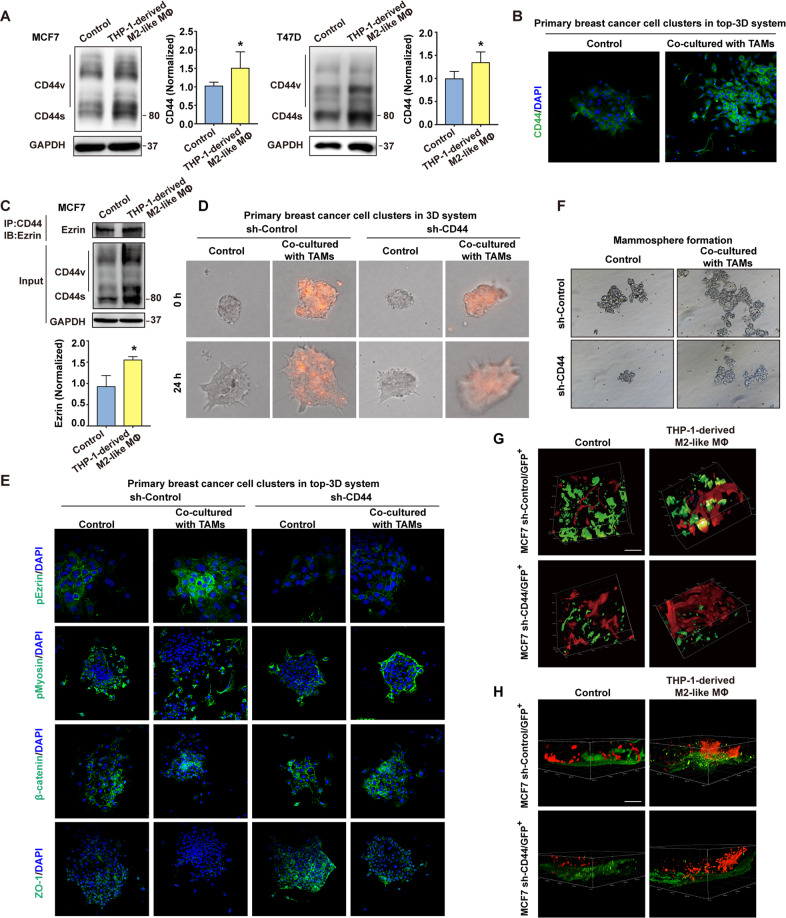
The essential role of CD44^high^ state and CD44-related invadopodia molecules in cohesive detachment. **A** Adaptive CD44 expression of luminal-like BrCa cells (MCF7 and T47D) upon THP-1-derived M2-like macrophages stimulation in a non-contact transwell system was assessed by immunoblotting analysis. GAPDH was used as control. The band intensities were analyzed by densitometry analysis. ^*^p < 0.05. **B** Representative confocal images of CD44 expression in disseminated cell clusters on top-3D matrix membrane during collective detachment. DAPI was used to stain the nuclei. An acquired CD44^high^ state upon TAMs stimulation in primary BrCa cell clusters (purified from MMTV-PyMT tumor) was observed by immunofluorescence analysis. **C** Co-immunoprecipitation experiment from whole-cell extracts demonstrating the interaction between CD44 and Ezrin after co-cultured with THP-1-derived M2-like macrophages in a non-contact transwell system. The band intensities were analyzed by densitometry analysis. ^*^p < 0.05. **D** The influence of CD44 knockdown on collective invasion of cell clusters in 3D matrix membrane. The sh-Control and sh-CD44 primary BrCa cell clusters, premixed with or without TAMs, were embedded in 3D system and recorded by time-lapse microscopy. TAMs were pre-stained with Vybrant CM-DiI (red). **E** Representative confocal images of localization of pEzrin, pMyosin, β-catenin and ZO-1 at the invasive and disseminating clusters. The sh-Control and sh-CD44 of primary BrCa cell clusters, premixed with or without TAMs, were cultured in top-3D basement. **F** Knockdown of CD44 inhibited TAMs-induced formation of mammosphere. The mammosphere-forming capacity of primary BrCa cells purified from MMTV-PyMT tumors was detected. Primary BrCa cells were directly sorted into wells of ultra-low attachment 24-well plate containing mammosphere growth medium, and then co-cultured with or without TAMs for 12 days in a non-contact transwell system. **G** Representative intravital images of subcutaneous xenografts derived from MCF7/sh-Control/GFP^+^ and MCF7/sh-CD44/GFP^+^ cells in vivo, which were injected with or without THP-1-derived M2-like macrophages, showing cohesive-invading MCF7/GFP^+^ cells approaching around vascular vessel. Tomato lectin (DyLight649, Cat. L32472, Thermo Fisher) was injected via tail vein to label vascular structures. Scale bars, 100 μm. See Supplementary Video S5–8. **H** Representative multiphoton confocal images of thick tumor tissues showed collective dissemination in subcutaneous xenografts. Tumor tissues were cut into 10- to 12-µm thick for paraffin sections, and the blood vessels were labeled by CD31 (Red channel) using immunohistochemical staining. Scale bars, 100 μm. See Supplementary Video S9–12.

We next investigated whether the CD44^high^ state and the CD44-associated pathway play a role in the cohesive shedding of BrCa cell clusters. Results showed that both CD44s and CD44v were increased at the protein level in MCF7 and T47D cells when co-cultured with THP-1-derived M2-like macrophages in a 2D system (Fig. 3A), illustrating the important role of TAMs in regulating CD44 alternative splicing. Similarly, CD44 was upregulated when MMTV-PyMT-derived BrCa cells were co-cultured with TAMs in the top-3D culture system (Fig. 3B). Given that the formation of CD44 with its intracellular downstream cytoskeleton proteins (Ezrin/Merlin) complexes has been implicated in the control of CD44-dependent directional cell motility [31], the interactions between CD44 and Ezrin/Merlin upon THP-1-derived M2-like macrophages stimulation were determined by co-immunoprecipitation assay. Our result found that, after co-cultured with THP-1-derived M2-like macrophages, the association between CD44 and Ezrin was enhanced (Fig. 3C), while the interaction between CD44 and Merlin was reduced (Fig. S3), indicating that an increased CD44-dependent cell motility was shown [31, 32].

To explore the role of the CD44^high^ state in collective detachment, primary cancer cell clusters transfected with sh-Control and sh-CD44 lentivirus were embedded with or without TAMs in 3D or top-3D basement membrane-rich gels. Time-lapse microscopy revealed that knockdown of CD44 suppressed TAMs-induced detachment of co-cultured clusters in 2D or 3D matrix membrane (Fig. 3D, Video S3–S4, and Fig. S4), supporting the hypothesis that CD44 acts as a vital effector in cohesive dissemination. In addition, in top-3D culture system, immunofluorescence assay revealed that the knockdown of CD44 inhibited the increase of TAMs-induced cohesive shedding and altered the localization of p-Ezrin, p-Myosin, β-catenin, and ZO-1, thus disrupting the disassembly of the adherent junction and tight junction triggered by TAMs (Fig. 3E). These results imply that CD44 is involved in the transition of clustered tumor cells to an invasive disseminating state induced by TAMs.

To further identify the contribution of CD44 to tumorigenic capability of the cell clusters induced by TAMs, we performed a mammosphere formation experiment. The results showed that the silence of CD44 significantly inhibited the TAMs-induced increase of mammosphere forming ability (Fig. 3F). To assess the role of CD44 in collective dissemination in vivo, we used intravital microscopy to analyze BrCa xenografts derived from MCF7/sh-Control/GFP^+^ and MCF7/sh-CD44/GFP^+^ cell clusters premixed with or without THP-1-derived M2-like macrophages. In tumors derived from MCF7/sh-Control cells premixed with THP-1-derived M2-like macrophages, the distribution of tumor cells was closer to blood vessels than that of MCF7/sh-Control cells only (Fig. 3G, H). However, the tendency of GFP^+^ cancer cells closing to blood vessels induced by THP-1-derived M2-like macrophages was significantly reduced in MCF7/sh-CD44 tumors (Fig. 3G, H, Video S5-12), suggesting that the knockdown of CD44 attenuated the collective detachment induced by TAMs in vivo.

### The critical role of TAMs-derived CCL8 in collective migration of tumor cells

**Fig. 4:**
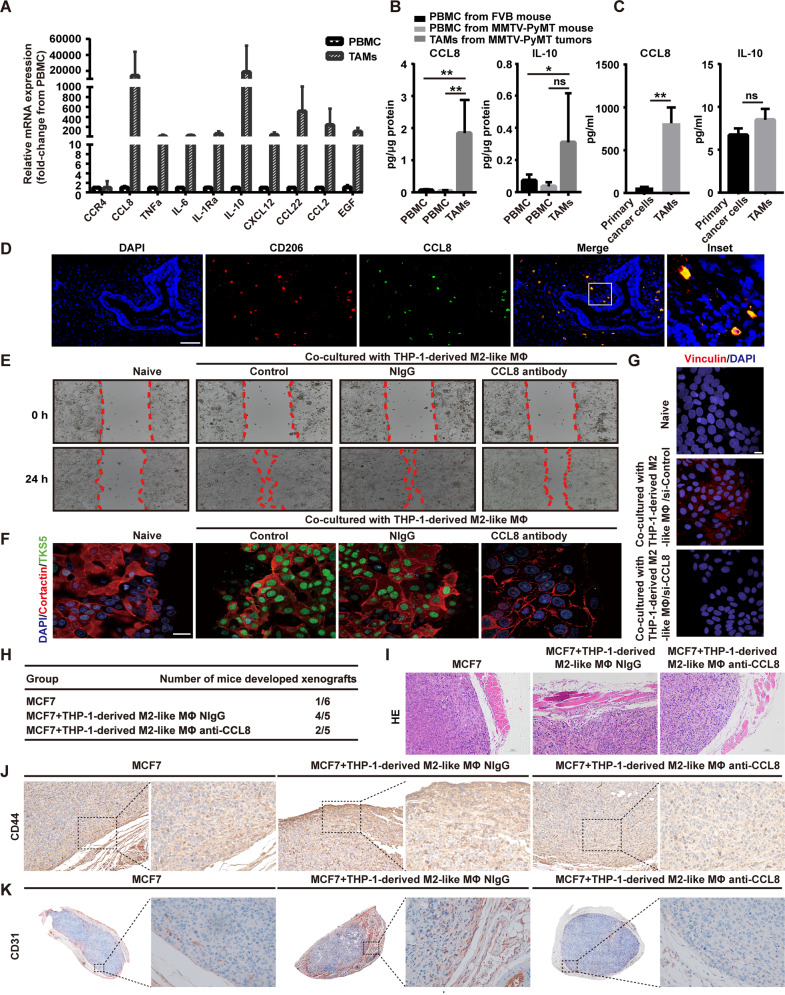
CCL8 derived from TAMs is associated with collective migration of tumor cells. **A** The mRNA levels of a panel of cytokines in TAMs versus PBMCs isolated from five primary breast tumors (MMTV-PyMT) were detected by qRT-PCR. **B** The intracellular CCL8 and IL-10 in TAMs versus PBMCs isolated from normal mouse or tumor-bearing mouse (MMTV-PyMT tumor) were determined by ELISA. *n* = 5/group. Bars correspond to mean ± SD. *p < 0.05, **p < 0.01, ns, nonsignificant. **C** The secreted CCL8 and IL-10 in TAMs versus primary BrCa cells were determined by ELISA. Cells were isolated from five primary breast tumors (MMTV-PyMT) and cultured separately. Bars correspond to mean ± SD. **p < 0.01, ns, nonsignificant. **D** Fluorescence in situ hybridization analysis of human BrCa tissue sections revealed that CCL8 mRNA is found in CD206^+^ TAMs but not in cancer cells. The expression of CD206 was detected using immunochemical staining. Inset representing a CD206^+^ macrophage-expressing CCL8 mRNA. Scale bars, 100 μm (*n* = 3). **E** Blocking of CCL8 inhibited the collective migration induced by THP-1-derived M2-like macrophages. In vitro scratch assay of untreated MCF7 or treated with THP-1-derived M2-like macrophages, THP-1-derived M2-like macrophages plus normal IgG, or THP-1-derived M2-like macrophages plus CCL8 antibody for the indicated period of time. Red line, cell culture margins (*n* = 4). **F** Blocking of CCL8 inhibited the re-distribution of invadopodia molecules in collective migration induced by THP-1-derived M2-like macrophages. Representative confocal images showed the re-distribution of Cortactin (red) and TKS5 (green) at the migrating front of clusters after CCL8 blocking for 3 days. MCF7 cells co-cultured with THP-1-derived M2-like macrophages in a non-contact transwell system, followed by treatment with normal IgG or CCL8 antibody. Scale bars, 20 μm. **G** Knocking down of CCL8 in THP-1-derived M2-like macrophages inhibited the increase of Vinculin at the migrating front of clusters. Wound healing assay was used to observe the changes of Vinculin expression at the migrating front. M2-like macrophages derived from THP-1 were transfected with siRNAs (si-Control or si-CCL8) and then co-cultured with MCF7 cells in a non-contact transwell system for 3 days. The expression of Vinculin was determined by immunofluorescence assay. Scale bars, 10 μm. **H** Blocking of CCL8 inhibited the onset of xenografts induced by THP-1-derived M2-like macrophages in vivo. MCF7 cells were orthotopically implanted into mammary pad of nu/nu mice, followed by intraperitoneal administration of a neutralizing antibody for CCL8. **I** HE staining of MCF7 xenografts in normal IgG or CCL8 antibody-treated mice. **J** CD44 (brown) expression of MCF7 xenografts in normal IgG- or CCL8 antibody-treated mice. **K** CD31 (red) expression of MCF7 xenografts in normal IgG- or CCL8 antibody-treated mice.

Given that TAMs are of heterogeneous nature, which exhibit tissue-specific phenotypes and gene signatures depending on tumor subtype [21], we identified the TAMs-derived factors in luminal-like BrCas. We performed investigations by quantitative RT-PCR (qRT-PCR) in paired CD206^+^ TAMs and peripheral blood monocytes (PBMCs), which were isolated from five MMTV-PyMT tumors and normal PBMCs obtained from five healthy FVB mice. Among the panel of cytokines related to TAMs, CCL8 and IL-10 are relative abundantly expressed (Fig. 4A). To confirm the CCL8 and IL-10 expressions in TAMs from BrCa, the intracellular (Fig. 4B) and secreted CCL8 and IL-10 protein levels (Fig. 4C) were determined by ELISA. Results showed that cytoplasmic CCL8 in TAMs was significantly higher than that of PBMCs from MMTV-PyMT tumors or healthy mice (Fig. 4B; *p* < 0.01). CCL8 production in the supernatant of cultured TAMs was also significantly higher than that of cancer cells from primary BrCas (Fig. 4C; *p* < 0.01). Furthermore, the amount of cytoplasmic or secreted CCL8 in TAMs was substantially higher than that of IL-10 (Fig. 4B, C). Additionally, fluorescence in situ hybridization investigation of human BrCa demonstrated that CCL8 mRNA is found in CD206^+^ TAMs rather than cancer cells (Fig. 4D), implying a possible effect of CCL8 produced by TAMs on primary tumors. Meanwhile, a positive correlation between CD206 and CCL8 mRNA levels in BrCa patients was discovered using the Cancer Genome Atlas (TCGA) database (Fig. S5), further confirming the importance of CCL8 in TAMs.

To verify the contribution of CCL8 in collective migration, an anti-CCL8 neutralizing antibody was used. As expected, blocking of CCL8 inhibited the increase of collective migration induced by THP-1-derived M2-like macrophages (Fig. 4E) and reduced the up-regulation of Cortactin and TKS5 in the leading edge of collective migrating sheets (Fig. 4F). In addition, in the lamellipodia of migrating primary BrCa cells, vinculin as a component of nascent adhesions [33], was up-regulated when co-cultured with THP-1-derived M2-like macrophages which could be inhibited after knocking down of CCL8 (Fig. 4G). Collectively, these data suggest that TAMs could promote cohesive migration of BrCa cells via CCL8.

To address whether CCL8 could induce the onset of cancer cell clusters, as predicted from in vitro model [21], we orthotopically implanted MCF7 cells with or without THP-1-derived M2-like macrophages into nu/nu mice and evaluated the onset changes of the tumor incidence upon blocking of CCL8. Neutralizing antibody of CCL8 or normal IgG was intraperitoneally administered daily for 5 days, respectively, following implantation of cancer cells. Results showed that the blocking of CCL8 activity significantly reduced THP-1-derived M2-like macrophages-induced tumorigenicity (Fig. 4H, I). Also, reduced CD44 and CD31 expressions were observed in CCL8 antibody-treated tumors (Fig. 4J, K), suggesting that the blocking of CCL8 reduced the THP-1-derived M2-like macrophages-induced increase of tumor onset and angiogenesis.

### The activation of MDM2-p53 signaling pathway in CD44^high^ state induced by CCL8

**Fig. 5:**
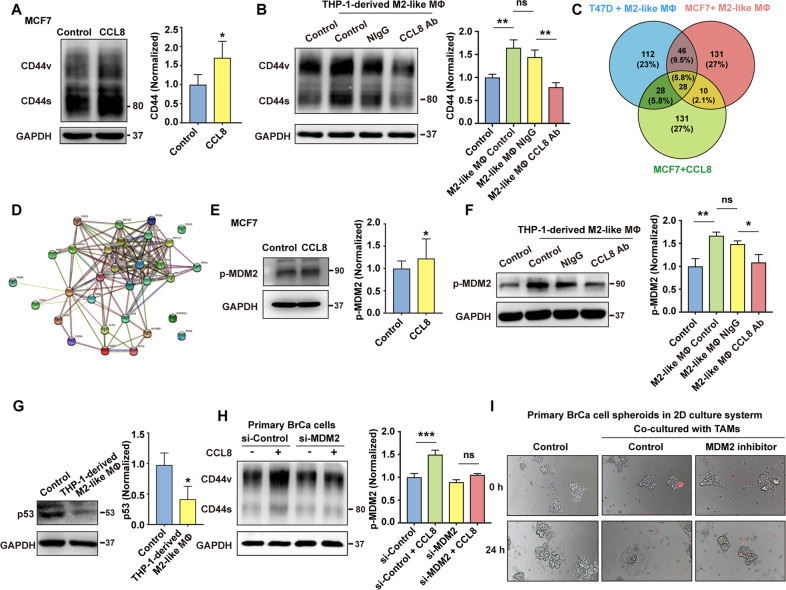
The activation of MDM2-p53 signaling pathway in CD44^high^ state induced by CCL8. **A** The influence of CCL8 on the expression of CD44 in MCF7 cells was evaluated by western blot. The band intensities were analyzed by densitometry analysis. ^*^p < 0.05. **B** Blocking of CCL8 inhibited the acquisition of CD44^high^ state in MCF7 cells induced by M2-like macrophages, which was obtained from human monocytic cell line THP-1 stimulated by PMA/m-CSF/IL-4/IL-10/IL-13. The band intensities were analyzed by densitometry analysis. ^**^p < 0.01, ns: no significance. **C** Scheme of identifying differential CD44-interacting peptides upon THP-1-derived M2-like macrophages/CCL8 stimulation. The intersection showed the CD44-interacting proteins, which were analyzed by co-immunoprecipitation-based mass spectrometry assay. MCF7 and T47D cells were stimulated by THP-1-derived M2-like macrophages or CCL8 for 3 days, respectively. The proteins recruited to CD44 were precipitated by CD44 antibody, and then subject to mass spectrometry assay. **D** Functional associations of the regulatory networks of MDM2/p53-correlated genes from analysis of STRING data are presented. **E** The effects of CCL8 on the activation of MDM2 in MCF7 cells were evaluated by immunoblotting assay. The band intensities were analyzed by densitometry analysis. ^*^p < 0.05. **F** Blocking of CCL8 inhibited the activation of MDM2 in MCF7 cells induced by THP1-derived M2-like macrophages. The band intensities were analyzed by densitometry analysis. ^*^p < 0.05, ^**^p < 0.01, ns: no significance. **G** Repression of p53 expression by M2-like macrophages in human BrCa cells was detected by immunoblotting assay. The band intensities were analyzed by densitometry analysis. ^*^p < 0.05. **H** Knocking down of MDM2 in primary BrCa cells inhibited the up-regulation of CD44 induced by TAMs. The band intensities were analyzed by densitometry analysis. ^***^p < 0.001, ns: no significance. **I** The influence of MDM2 inhibitor on collective dissemination of cell clusters induced by TAMs in 2D culture system. Before co-cultured with TAMs on 2D culture system, primary BrCa cell clusters were obtained from an ultra-low attachment culture system. TAMs were pre-stained with Vybrant CM-DiI (red).

To further identify the details of CCL8 function in CD44^high^ state induced by TAMs, an exogenous recombinant CCL8 was added to the culture medium of MCF7 cells. The results showed that CCL8 significantly upregulated the expression of CD44 (Fig. 5A). Further, the CCL8 neutralizing antibody inhibited the THP-1-derived M2-like macrophages-induced CD44 activation in BrCa cells (Fig. 5B). These data demonstrated that M2-like macrophages-derived CCL8 is one of the critical cytokines that increase the expression of CD44 in BrCa cells. Next, we used a CD44-dependent co-immunoprecipitation assay-based mass spectrometry to compare peptides in MCF7 and T47D cells that were differently regulated by THP-1-derived M2-like macrophages or CCL8. By analyzing the differential peptides immunoprecipitated by CD44 antibody, we found that THP-1-derived M2-like macrophages activation caused an overexpression of several intracellular signaling molecules closely associated with MDM2/p53-signaling pathway (Fig. 5C, D, and Supplementary Data 2). However, it remains unknown whether MDM2 or p53 is responsible for driving the CD44-dependent detachment induced by THP-1-derived M2-like macrophages.

Therefore, to explore the effects of TAMs or CCL8 on MDM2/p53 signaling activation, the expression of p-MDM2 and p53 was detected. The results showed that neutralizing antibody of CCL8 inhibited the increase of phosphorylation of MDM2 induced by THP-1-derived M2-like macrophages or CCL8 (Fig. 5E, F). Meanwhile, the expression of p53 was depressed by THP-1-derived M2-like macrophages (Fig. 5G). Given that MDM2 is thought to inhibit p53 activity and enhance its degradation [34, 35], we next investigated the role of MDM2 in TAMs-induced CD44 activation. Our results showed that the knockdown of MDM2 significantly reduced the up-regulation of CD44 induced by CCL8 (Fig. 5H), suggesting that CD44 expression was controlled by MDM2/p53 signaling pathway after TAMs stimulation. Finally, we verified the role of MDM2/p53 signaling in CD44-dependent collective detachment and found that the MDM2 inhibitor significantly suppressed the BrCa cells collective dissemination (Fig. 5I).

### CD44-Ezrin-p38 pathway in collective detachment induced by TAMs

**Fig. 6:**
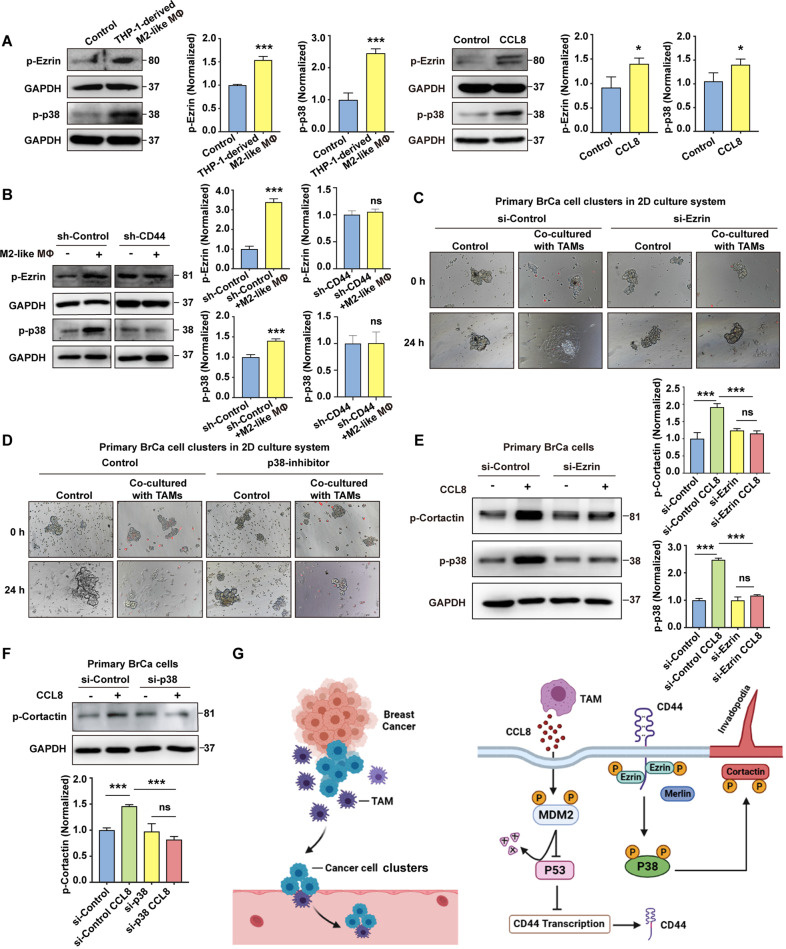
The CD44-Ezrin-p38 pathway in collective detachment induced by TAMs. **A** Activation of Ezrin and p38 after MCF7 cells co-cultured with THP-1-derived M2-like macrophages or CCL8 by a non-contact transwell system was determined by immunoblotting assay. The band intensities were analyzed by densitometry analysis. ^*^p < 0.05, ^***^p < 0.001. **B** Knocking down of CD44 inhibited the activation of Ezrin and p38 induced by THP-1-derived M2-like macrophages or CCL8. The band intensities were analyzed by densitometry analysis. ^***^p < 0.001, ns: no significance. **C** Knocking down of Ezrin in primary BrCa cells inhibited collective dissemination induced by TAMs in 2D culture system. The primary BrCa cell clusters were transfected by si-Ezrin. TAMs were pre-stained with Vybrant CM-DiI (red). **D** The p38 inhibitor attenuated collective dissemination of primary BrCa cell clusters induced by TAMs. TAMs were prestained with Vybrant CM-DiI (red). **E** Knocking down of Ezrin inhibited phosphorylation of Cortactin and p38 triggered by CCL8. The band intensities were analyzed by densitometry analysis. ^***^p < 0.001, ns: no significance. **F** Knocking down of p38 repressed the phosphorylation of Cortactin induced by CCL8. The band intensities were analyzed by densitometry analysis. ^***^p < 0.001, ns: no significance. **G** Scheme summarizing the proposed mechanism by which MDM2-p53-p38 signaling pathway mediated the acquisition of CD44^high^ state, which leading to cohesive detachment.

Given that Ezrin phosphorylation necessitates the activation of the p38 MAP kinase [36], we further investigated whether CD44-Ezrin complexes could induce cancer cells dissemination through p38 activation. As shown in Fig. 6A, B, a rapid phosphorylation of p38 and Ezrin was observed in BrCa clusters after THP-1-derived M2-like macrophages or CCL8 stimulation, which was suppressed by knocking down of CD44. Moreover, knocking down of Ezrin and treating with the p38 inhibitor significantly reduced TAM-induced dissemination of cancer cell clusters (Fig. 6C, D). The data suggested that Ezrin/p38 signaling mediated by CD44 was involved in collective dissemination. Based on these results, we hypothesized that CCL8 may trigger invadopodia formation through Ezrin and p38. Indeed, the knocking down of Ezrin in primary BrCa cells markedly attenuated the effect of CCL8 on phosphorylation of Cortactin and p38 (Fig. 6E). Meanwhile, the activation of Cortactin induced by CCL8 was inhibited by p38 knocking down (Fig. 6F).

Collectively, our results suggested that CCL8 derived from TAMs in breast cancer may activate MDM2, which attenuates the expression of p53, thus giving rise to CD44 activation. In addition, the interaction between CD44 and Ezrin-activated p38 signaling may induce the formation of invadopodia (summarized in Fig. 6G).

## Discussion

Metastasis is the main cause of death in breast cancers (BrCas), during which cancer cells or collective clusters disseminating from tumor mass is the critical step. However, the underlying mechanism is still obscure. Our study uncovered a novel mechanism of the clustered cancer cells in the onset of the collective dissemination. Notably, we identified the critical role of a CD44^high^ state in triggering BrCas cohesive detachment, which was induced by tumor-associated macrophages (TAMs).

CD44, a non-kinase glycoprotein, is ubiquitously expressed on various types of cells including cancer stem cells (CSCs) and has been widely studied in recent years in cancer onset and aggressiveness. As a transmembrane receptor, CD44 interacts with its downstream cytoskeletons such as ERM (Ezrin, Radixin, and Moesin) family proteins in a reversible manner, resulting in changes in cell motility, adhesion, and invasion by triggering a series of intracellular signal cascades. In addition, CD44 can mediate intercellular communications which contribute to tumor cell aggregation, in the way similar to other adhesion molecules such as E-cadherin [37, 38] and PECAM1 [39, 40]. As our laboratory showed before, CD44 marks highly invasive epithelial cell populations in BrCas [8] and shows a high expressing potential in metastases sites [41]. On the contrary, CD44 expression is low in luminal-like BrCa with a weak invasive potential [8]. However, CD44 could be found to be highly expressed at the invading edges in the luminal-like BrCas, suggesting that CD44 is activated during collective invasion. Data have shown that in experimental models, changes in genes like BRCA1 [42, 43], cytokeratin-14 [6, 24], and Met [44], could lead to the shift of luminal phenotype towards basal invasive features. Consistent with these observations, several studies have also shown that CD44 is linked to epithelial to mesenchymal transition (EMT) and the switch of non-CSC to CSC [12, 45–47]. Our present study provides an additional support to these findings showing that CD44 is involved in the initiation of collective detachment from cohesive invasion.

Importantly, in this study, we found that although disseminated cancer cell clusters displaying highly expressed CD44 were mobile and invasive, the cell-cell junction molecules were not lost (e.g., Msn, Fn1). Indeed, analysis of the gene expressing signatures in the disseminated subpopulation by RNA sequencing identified a cohort of genes related to partial EMT, invasiveness, and maintenance of cell-cell contact. These data suggested that the CD44^high^ state could cause collective detachment rather than individual tumor cells dissemination. In 3D organotypic culture, we also found a CD44-dependent redistribution of invadopodia molecules after TAMs stimulation, such as p-Ezrin, p-Myosin, and β-catenin which connect to an increased capability of invasion. Actually, we previously reported that Ezrin, one of the ERM family proteins, was involved in CD44-dependent collective invasion [8]. In this study, we further observed that Ezrin could interact with CD44 to increase the cohesive detachment. Therefore, it is reasonable to speculate that the CD44-Ezrin interactions may promote BrCa cell clusters motility at the leading edge accompanied by the reduced coordination between adjacent cells, which eventually results in cohesive cell detachment.

Cancer cells motility and invasion are crucial for metastatic initiation, which is significantly influenced by tumor microenvironment (TME) including cellular and non-cellular components that may vary depending on the tumor subtypes. TAMs are key components of stromal cells in TME which play critical roles on the invasion of cancer cells and usually show distinct phenotypes depending on tumor-specific signatures [21, 48, 49]. For example, different phenotypes of TAMs can release different cytokines depending on molecular subtypes of BrCas. Although accumulating evidence has demonstrated that the direct contact between macrophages and tumor cells can lead to the extravasation of single tumor cells into blood vessels [50], little is known about the effects of TAMs on cancer clusters behaviors. We previously reported that CD44 is dynamically regulated by the components of TME during collective invasion [8]. In the present study, we demonstrated that CD44 initiated collective detachment and its associated signaling could be activated by TAMs. As the function of TAMs on tumor metastasis is largely dependent on cytokines released, we next showed that CCL8 is responsible for the movement of cell clusters induced by TAMs in luminal-like BrCas. Although we cannot exclude other cytokines in the TAMs induced collective detachment, our study provided direct evidence for the vital role of TAMs in cohesive dissemination.

Further, our mechanistic results revealed that an MDM2/p53 signaling cascade activated by CCL8 was involved in the initiation of the CD44-induced cohesive detachment. As previous reports have proved that CD44 can be repressed by p53, and p53 can be degraded by MDM2 [10, 35, 51], our finding that MDM2 signaling could regulate CD44 expression reveals a new mechanism that connects MDM2 with CD44 in tumor collective detachment. Meanwhile, we also found that the downstream of MAPK/p38 was involved in CD44-dependent cohesive detachment, implying that the MDM2-p53-CD44-p38 axis may be responsible for cortical movement in luminal BrCa cell clusters detachment. Future studies are needed to uncover the inside details of the mechanisms.

In summary, we have elucidated a novel finding that the collective detachment of luminal-like BrCas could be mediated by the CD44^high^ state. Moreover, our results also demonstrated that TAMs were responsible for the CD44 activation which initiated a series of downstream signaling cascades. Future research is warranted to focus on BrCa cell clusters in collective metastasis for therapeutically targeting.

## Materials and methods

### Cell culture and mice

The human BrCa cell lines MCF7 and T47D were purchased from the American Type Culture Collection (ATCC). MCF7 cells were cultured in MEM (Gibco, USA), and T47D cells were cultured in high-glucose DMEM (Gibco, USA). All the media were supplemented with 10% fetal bovine serum and 100 IU/ml penicillin/streptomycin. Additional insulin with a final concentration of 0.01 mg/ml was added to the culture medium of MCF7. All cell lines were cultured at 37 °C in humidified air with 5% CO_2_ and tested for mycoplasma contamination. In this study, MMTV-PyMT (FVB/N) mice were obtained from Mode Animal Research Center of Nanjing University.

### Antibodies and reagents

Primary antibodies used were listed in Supplementary Table 2. The p38 inhibitor (SB202190) was purchased from MedChemExpress (Monmouth Junction, NJ, USA). MDM2 inhibitor Nutlin-3 was obtained from Selleckchem (Houston, TX, USA). Ezrin inhibitor (NSC668394) was purchased from EDM Millipore (Danvers, MA, USA). Recombinant human CCL8 (MCP-2) was obtained from Biolegend (San Diego, CA, USA).

### Patients and tissue samples

To test whether TAMs present in circulating multicellular cluster in BrCas, we evaluated human breast biopsies using a small cohort (15 luminal-like BrCas) from patients who underwent mastectomy at Shanghai Sixth People’s Hospital. Patients who received chemotherapy or radiotherapy before surgery were excluded. A tissue microarray (85 human breast lesions) obtained from Shanghai Superchip Biotech (HBreD090Bc02) was used to analyze the expression of CD44 or CD206 between tumors with and without microemboli groups. Informed consent was obtained from all subjects. Clinicopathological characteristics of the BrCa patients, including age, tumor size, histologic grade, lymph node metastasis, and tumor thrombus, were obtained by reviewing medical charts and pathology records (Supplementary Table 1).

### Immunohistochemical staining of tumor samples

Primary mammary tumors from MMTV-PyMT mice and primary breast cancer samples from BrCa patients were extracted and formalin-fixed, followed by paraffin embedding. For immunostaining, paraffin sections were fixed in acetone, air dried, and then stored at −80 °C. Tumor sections were dewaxed with xylene and dehydrated in gradient ethanol, and then the antigen was recovered in citrate antigen recovery solution. Endogenous peroxidase activity was eliminated with endogenous peroxidase blocking buffer for 20 min. Then, tissues were replenished with PBS and blocked with 3% BSA in PBS for 1 h. Subsequently, sections were treated with primary antibodies (CD206, CD44, CD31, CK) overnight at 4 °C. After incubation with horseradish peroxidase (HRP)-conjugated secondary antibody for 1 h at room temperature, the slides were stained with diaminobenzidine for 1 h at room temperature, followed by counterstaining with hematoxylin. The staining was visualized using a light microscope.

### FACS analysis and sorting

Breast tumors were isolated from MMTV-PyMT mice, and then the tumor tissues were digested into single-cell suspension according to the protocol of tumor dissociation kit (Miltenyi, Cat No. 130-096-730). Cells were harvested and washed with Hanks’ balanced salt solution (HBSS) for three times. Then, the cells were resuspended with 100 μl HBSS. F4/80-APC (Invitrogen, Cat No. 17-4801-82), CD206-PE (Invitrogen, Cat No. 321106), CD45-FITC (Invitrogen, Cat No. 11-0451-82), EpCAM-PE-Cyanine7 (Invitrogen, Cat No. 25-5791-80) were added and incubated on ice for 30 min. After incubation, stained cells were washed by excess unbound antibodies and resuspended in HBSS. EpCAM^−^/CD45^+^/F4/80^+^/CD206^+^ or EpCAM^+^/CD45^−^/F4/80^−^/CD206^−^ subpopulations were sorted from MMTV-PyMT tumors by FACS.

### Three-dimensional Matrigel culture

MMTV-PyMT cancer cells and TAMs sorted by flow cytometry were pre-cultured in ultralow attachment 24-well plates, respectively, and microspheres were formed after 24 h. M2 macrophages were stained with CM-Dil (Invitrogen, Cat No. v22888). Then DMEM medium containing 50% Matrigel (BD, Cat No. 354234) and a final concentration of 4 mg/ml collagen-I (BD, Cat No. 354249) were prepared. The pH was subsequently adjusted using 1 mol/L NaOH. The pre-cooled 96-well plates were precoated by adding 30 μl medium with collagen/Matrigel to each well, followed by incubating at 37 °C for 30 min to let the collagen/Matrigel solidify. Then the MMTV-PyMT cancer cells and TAMs microspheres were added into the precoated 96-well plate separately or together, centrifuged at 100 rpm for 5 min, and the supernatant was discarded. Finally, 80 μl medium with collagen/Matrigel was covered on microspheres for 3D culture, or 100 μl medium was added for top 3D culture at 37 °C and 5% CO_2_.

### Living images of cell migration assay

The cells were imaged using Nikon Living Cell Observation system with a confocal microscope (Nikon A1, Tokyo, Japan). In general, images were collected every 30 min. The exposure time was 250 ms and several movies were recorded. The cells were maintained at 37 °C and 5% CO_2_.

### Mice

To study the effect of TAMs on tumor growth and metastasis in vivo, we sorted MMTV-PyMT cancer cells and TAMs for subcutaneous tumorigenesis experiments. Female BALB/c nude mice (5–6 weeks old) were randomly divided into two groups according to body weight. Cancer cell clusters (n = 6) were inoculated with 0.2 ml mixture of PBS and Matrigel at a ratio of 1:1 containing cancer cells (5 × 10^5^ cells) with or without TAMs. The mixture of MMTV-PyMT cancer cells and TAMs (5 × 10^5^ cells) was at a ratio of 1:1. The investigator was not blinded to the group allocation during the experiment. Tumor size was measured by caliper. Mice were sacrificed 40 days later. Tumor and lung tissues were isolated, fixed with formalin, and embedded in paraffin for HE staining.

For animal intravital two-photon microscope imaging, female nude mice (5–6 weeks old) were randomly divided into four groups. MCF7 cells were engineered to express the green fluorescent protein gene (GFP). MCF7 sh-Control/GFP^+^ and MCF7 sh-CD44/GFP^+^ cells (1 × 10^6^ cells, *n* = 3) were inoculated with 0.2 ml mixture of PBS and Matrigel at a ratio of 1:1 with or without THP-1-derived M2-like macrophages.

To address whether CCL8 induced the onset of cancer cell clusters, female nude mice (5–6 weeks old) were randomly divided into three groups. Mice were inoculated with 0.2 ml mixture of PBS and Matrigel at a ratio of 1:1 containing MCF7 cells only (4 × 10^6^/mouse, *n* = 6) or MCF7 cells mixed with THP-1-derived M2-like macrophages at a ratio of 9:1. Six days later, the mice were inoculated with THP-1-derived M2-like macrophages followed by daily intraperitoneal injections of anti-CCL8 antibody (*n* = 5) or normal IgG (*n* = 5).

All protocols involving mice were evaluated and approved by the Institutional Animal Care and Use Committee of Jiao Tong University Affiliated Sixth People’s Hospital and performed under veterinary supervision.

### Microdissection

MMTV-PyMT cancer cells were cultured in low attachment 96-well plate for 24 h to generate cell clusters. Then cell clusters were mixed with TAMs and incubated at foil bottom dishes (Willco Wells, Cat No. FWST-5030-4) to study microdissection with ‘Safe Grip’ rim. The dishes were pre-coated with Matrigel/Collagen-1 Matrix gel. After spreading on the Matrigel/Collagen-1 Matrix gel for 24 h, we used the Leica LMD microdissection system to isolate the disseminating cells of the cell clusters. Briefly, disseminated leader cells and the inner cells were dissected, respectively. Microdissected cells with foil bottom were fell by gravity into a sterile 1.5 ml Eppendorf tube. Approximately 100 single cells in tube were collected for RNA-sequencing.

### RNA sequencing

RNA was extracted from the disseminated leader cells or the inner cells using phenol-chloroform extraction for Illumina RNA sequencing analysis (Illumina HiSeq X-Ten). RNA sequencing library was prepared using an Illumina TruSeq RNA Sample Preparation Kit. Sequencing was performed on the Illumina HiSeq instrument.

### Quantitative real-time PCR

Total RNA was extracted from cultured cells or frozen tissues using Trizol reagent (Takara, Dalian, China). RNA samples were reverse transcribed to complementary DNA (cDNA) using a cDNA Synthesis Kit (Takara, Dalian, China). cDNA was amplified with a SYBR Green PCR Kit (Takara, Dalian, China) on an ABI 7500 system (The primers used were listed in Supplementary Table 3). Glyceraldehyde-3-phosphate dehydrogenase (GAPDH) was amplified as an internal control. The quantitative real-time PCR analysis was performed in triplicates.

### Co-culture procedure

THP-1 monocytes (2 × 10^6^ cells/ml) were incubated into 25 cm^2^ cell culture flasks with 320 nmol/L PMA for 24 h in RPMI 1640 complete medium. Then the medium was replaced by fresh medium supplemented with 25 ng/ml hm-CSF for 6 days and the culture medium was changed ever three days. Subsequently, the cells were incubated with 20 ng/ml IL-4, IL-10, and IL-13 for 24 h to generate THP-1-derived M2-like macrophages. MCF7 cells (5 × 10^5^ cells/well) and THP-1-derived M2-like macrophages (3 × 10^5^ cells/well) were co-cultured in 24 well plates (Corning, NY, USA) with 0.4 μm porous membrane to separate the upper and lower chambers. Then MCF7 cells in lower chambers were collected for western blot or quantitative real-time PCR.

### Western blot

Thirty micrograms of protein were separated by sodium dodecyl sulfate-polyacrylamide gel electrophoresis (SDS-PAGE) and then imprinted onto a polyvinylidene fluoride (PVDF) membrane. The transferred membrane was blocked in skimmed milk for 1 h and subsequently incubated with primary antibodies at 4 °C overnight. After incubation with primary antibodies, the membranes were washed and incubated with HRP-conjugated secondary antibodies, and developed using standard enhanced chemiluminescence (ECL) system. The signal was captured by Amersham Imager 680 (GE Healthcare, Madison, Wisconsin, USA). All procedures were performed in triplicates. Uncropped immunoblot gels are shown in Supplementay data 3.

### Immunofluorescence

Cells were fixed in 4% paraformaldehyde for 10 min, permeabilized with Triton X-100/phosphate-buffered saline (PBS), and blocked in 1 × PBS + 1% bovine serum albumin (BSA). Cells were incubated with primary antibodies overnight at 4 °C in 1 × PBS + 1% (BSA) and then with secondary antibodies conjugated with Alexa Fluor 488 or 647 (Abcam, Cambridge, MA, USA) for 1 h at room temperature. Images were analyzed under confocal microscopy (Nikon A1, Tokyo, Japan).

### Co-immunoprecipitation

RIPA buffer (Beyotime, China) containing protease inhibitors and phosphatase inhibitors was used for protein extraction. For co-immunoprecipitation, 30 μl of lysate protein with A/G agarose beads and normal IgG antibodies (2–3 μg) were incubated for 2 h at 4 °C. Then the mixed lysate was centrifuged at 300 g for 3 min at 4 °C, the nonspecific proteins bound by normal IgG antibody and beads were centrifuged and precipitated, and the supernatant was transferred to a new 1.5 ml Eppendorf tube with 30 μl of protein A/G agarose beads and primary anti-CD44 antibody (2–3 μg). Then the mixture was incubated at 4 °C overnight. After washing by PBS buffer for three times, the immunoprecipitation complexes were detected by western blot with targeted anti-Ezrin or anti-Merlin antibodies.

### shRNA-mediated gene knockdown

The mouse CD44 shRNA lentiviral particles (sc-35534-V) were purchased from Santa Cruz (Dallas, TX, USA). Human CD44 shRNA (pLKD-CMV-U6-shRNA/CD44) targeting all CD44 isoforms, and control GFP shRNA lentiviral particles were obtained from Obio (Shanghai, China). Stably transfected cells were purified by FACS with a GFP marker.

### Mammosphere formation

Sphere formation was carried out in ultra-low adhesion plate (Corning), and DMEM/F-12 (Invitrogen, New York, USA) was supplemented with B27 (2%), basic fibroblast growth factor (10 ng/ml), and epidermal growth factor (20 ng/ml). MMTV-PyMT tumor-derived cancer cells were inoculated and cultured at a density of 2000 cells/well at 37 °C and 5% CO_2_ for 24 h. Inhibitor or transfection reagent was added during this period. After that, the sphere (>50 μm) was examined at 40× magnification using an Olympus microscope.

### Live animal intravital two-photon microscope imaging

Lycopersicon Esculentum (Tomato) Lectin (LEL, TL) and DyLight 649 (Invitrogen, New York, USA) were injected intravascularly in an attempt to label vascular elements in mice. Image data for tumors and blood vessels were collected by intravital two-photon microscopy (Nikon) on the anesthetized live mouse. Intravital imaging was performed on a Nikon two-photon microscope with a 25×, Apo LWD/1.10 W water immersion objective; a pulsed laser system with a wavelength range of 820-1300/1040 nm was used for excitation of fluorophores. GFP and Cy5/deep-Red were viewed simultaneously with 820 nm excitation, along with 500–550 nm and 601–657 nm emission filters for GFP and Cy5/deep-Red, respectively. During the imaging, the temperature of the mouse was monitored and maintained at 37 °C.

### Migration

MMTV-PyMT cancer cells alone or mixed with TAMs were pre-cultured in ultralow attachment 24-well plates to form microspheres for 24 h. Then the microspheres were removed and reinoculated in 35 mm imaging dish with an imprinted 500 µm cell location grid (ibidi, Cat No. 80156) to observe the spreading behavior of MMTV-PyMT cancer cells co-cultured with or without TAMs. During this period, the spreading behavior of cells can be observed by silencing CD44 or Ezrin, or inhibiting p38 activation.

### Wound healing

MCF7 cells were co-cultured with or without THP-1-derived M2-like macrophages, treated with normal IgG or CCL8 blocking antibody, and followed by seeding on two-well silicone insert (ibidi). The culture-insert was removed to generate a 0.5 mm wound gap when cells formed 100% confluent monolayers. A total of 3 × 10^4^ cells were seeded into each well with 80 µl of cell culture medium and incubated at 37 °C in a 5% CO_2_ humidified incubator for 24 h.

### Co-immunoprecipitation assay-based mass spectrometry

MCF7 and T47D cells were stimulated by THP-1-derived M2-like macrophages or CCL8 for 3 days, respectively. Then the immunocomplexes recruited to CD44 were obtained by co-immunoprecipitation assay with CD44 antibody. The immunocomplexes were washed three times with NP40 and analyzed by mass spectrometry assay. Briefly, samples were lysed in 8 mol/L urea and 100 mmol/L Tris solution (PH 7.6) after reduction of dithiothreitol and alkylation by iodoacetamide. The protein solution was digested by trypsin at 37 °C for 18 h. Then peptide solution was transferred to Solid Phase Extraction Cartridge for desalting and clean-up of sample. The samples obtained above were analyzed by a Q Exactive *HF-X* mass spectrometer (Thermo Fisher, San Jose, CA, USA) equipped with a Nanospray Flex source (Thermo Fisher). Samples were separated by a home-made micro-tip C18 column (75 mm × 200 mm) packed with ReproSil-Pur C18-AQ, 3.0 mm resin (Dr. Maisch GmbH, Germany) on a nanoflow HPLC Easy-nLC 1200 system (Thermo Fisher). The MS1 full scan was set at a resolution of 60,000 @ m/z 200, followed by ‘top 20’ MS2 scans generated by HCD fragmentation at a resolution of 15,000 @ m/z 200. The normalized collision energy (NCE) was set at NCE 28%, and the dynamic exclusion time was 45 s. All mass spectrometric data were analyzed using MaxQuant against the human UniProt database containing 192,656 sequences (08/2020). Carbamidomethyl cysteine was searched as a fixed modification. Oxidized methionine and protein N-term acetylation were set as variable modifications. Enzyme specificity was set as trypsin. The maximum missing cleavage site was set as 2. The tolerances of first search and main search for peptides were set at 20 ppm and 4.5 ppm, respectively. The minimal peptide length was set at 7. The false discovery rates (FDR) of peptide, protein and site were all <0.01.

### Statistical analysis

Statistical analysis was performed using GraphPad software. For normally distributed data, the analysis between two groups was performed using unpaired two-tailed Student’s *t*-test. The differences in CD206, CD44 and CD31 were analyzed by nonparametric Mann-Whitney tests. One-way ANOVA was used to evaluate significant differences between multiple comparison groups. The variance between the groups being statistically compared was similar. Statistical significance was defined as *p* < 0.05.

## Supplementary information

Supplementay data 1

Supplementay data 2

Supplementay data 3

Video S1

Video S2

Video S3

Video S4

Video S5

Video S6

Video S7

Video S8

Video S9

Video S10

Video S11

Video S12

a Reproducibility checklist

## Data Availability

The datasets generated and analyzed during the current study are available from the corresponding author on reasonable request.
