# Supplementary material for: Activation of CD44 signaling in leader cells induced by tumor-associated macrophages drives collective detachment in luminal breast carcinomas
Source: Cell Death Dis. 2022 Jun 9;13(6):540. doi: 10.1038/s41419-022-04986-4 (PMC9184589; doi:10.1038/s41419-022-04986-4)
Supplement: Supplementary file 1 — Supplementay data 1 [file 41419_2022_4986_MOESM1_ESM.docx]

**Supplementary Figures and Legends**

**
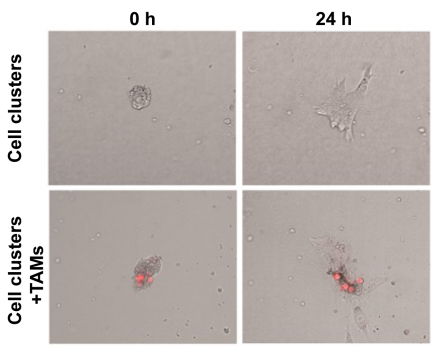
**

**Fig.S1 Cohesive shedding of primary BrCa cells in top-3D basement membrane-rich gels**

Primary cancer cells were co-cultured with TAMs from MMTV-PyMT tumors in top-3D collagen matrix for 24 h. Images represent 3 independent experiments. TAMs were pre-stained with Vybrant CM-DiI (red).

_
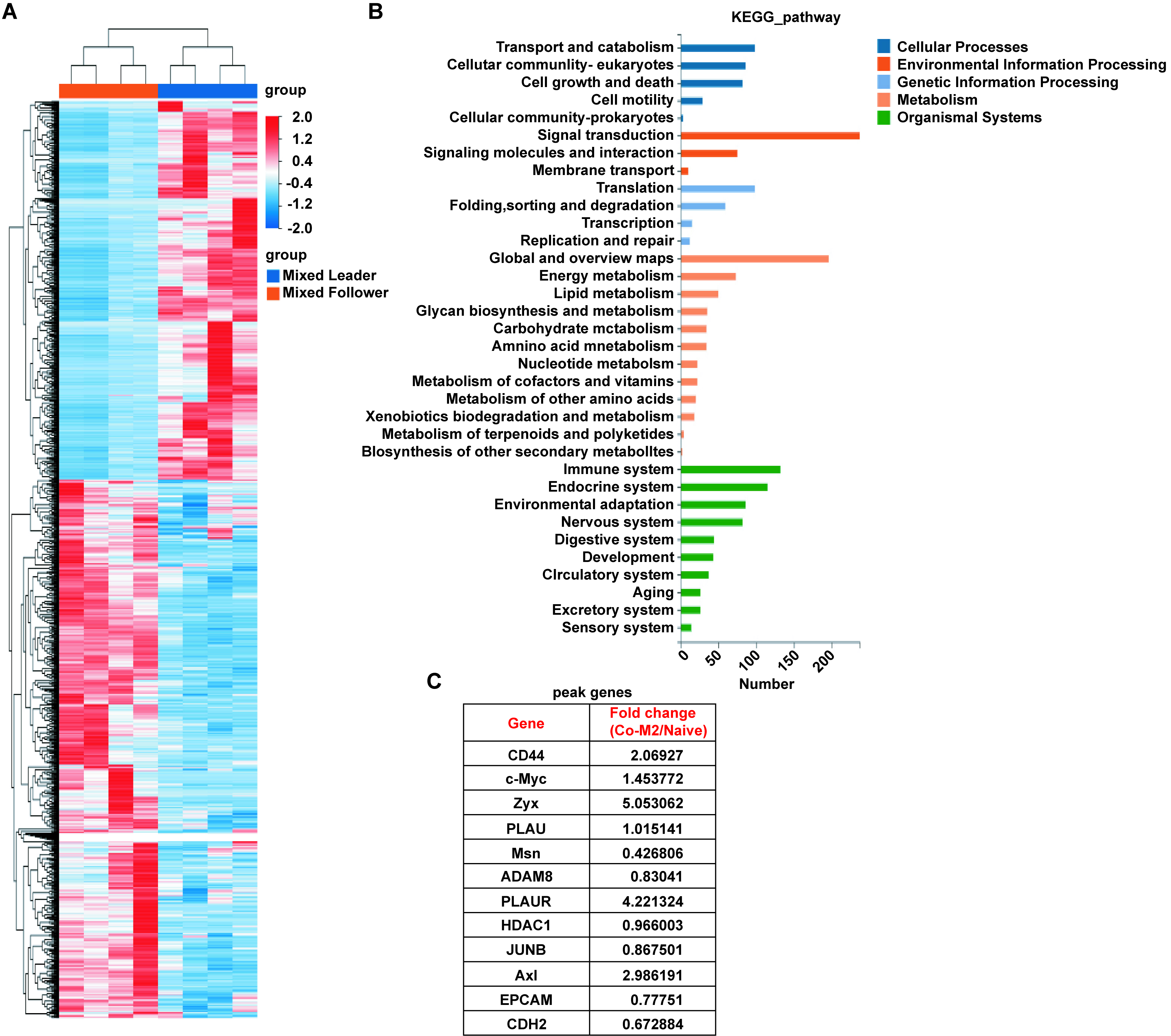
_

**Fig. S2 Gene expression of purified disseminated cell clusters and the inner cells in cell clusters.**

**a.** Unsupervised hierarchical clustering of 1753 significantly differentially expressed genes in

shedding cell clusters and the inner cells in cell clusters. **b.** KEGG analysis of gene expression differences in the disseminated cell clusters and the inner cells in cell clusters. **c.** The most differentially up-regulated transcripts in disseminated cells was verified by quantitative RT-PCR in MCF7 cell clusters co-cultured with THP-1-derived M2-like macrophages, compared with MCF7 cell clusters only, GAPDH was used as a loading control. Data are representative of at least three experiments.


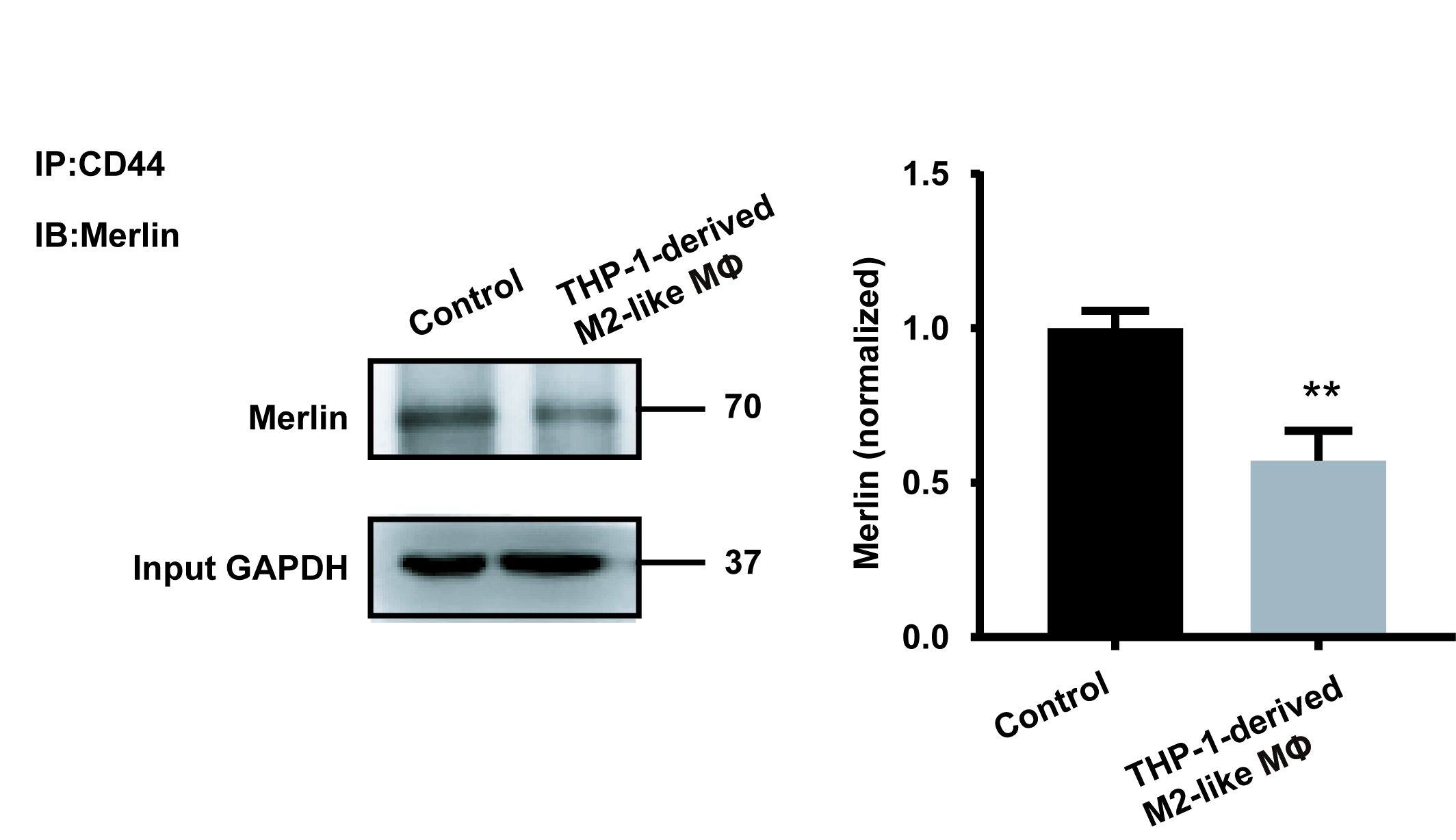


**Fig. S3 The association between CD44 and Merlin upon THP-1-derived M2-like macrophages stimulation**

Co-immunoprecipitation experiment from whole cell extracts demonstrating interaction between CD44 and Merlin after co-cultured with THP-1-derived M2-like macrophages in a non-contact transwell system. The interaction between CD44 and Merlin is decreased after co-cultured with THP-1-derived M2-like macrophages.


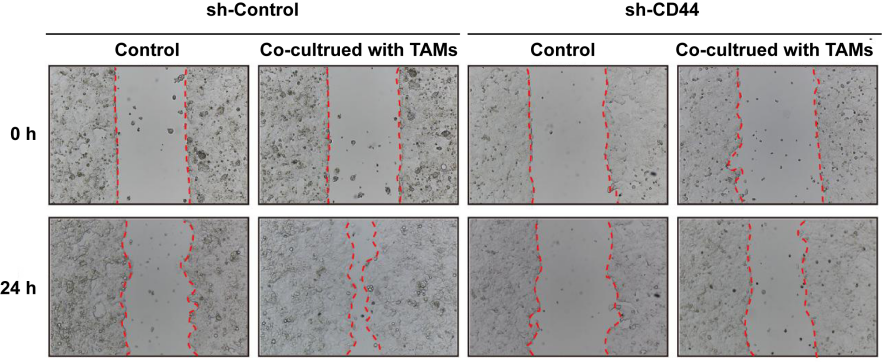


**Fig. S4 The influence of CD44 knockdown on collective migration induced by TAMs**

The primary BrCa cell clusters (sh-control and sh-CD44), premixed with or without TAMs, were cultured in 2D culture system and the collective migration was recorded by microscopy imaging.

**
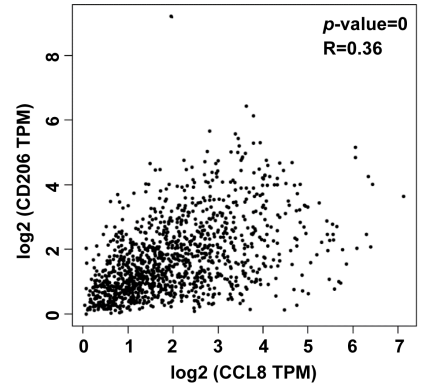
**

**Fig. S5 A positive correlation between CD206 and CCL8 mRNA levels in BrCa patients.** **Raw data were obtained from TCGA.**

**Supplemental Videos Legends**

**Movie 1:** (Related to Fig.2B) Time lapse imaging of the collective invasion of primary cell clusers from 18h to 36h.

**Movie 2:** (Related to Fig.2B) Time lapse imaging of the cohesive shedding of primary cell clusers co-cultured with TAMs (Red channel) from 18h to 36h.

**Movie 3:** (Related to Fig.3D) Collective invasion of primary BrCa cell clusters (sh-Control) embedded in 3D basement culture system, premixed with TAMs (Red channel), was recorded by time-lapse microscopy from 0h to 24h.

**Movie 4:** (Related to Fig.3D) Collective invasion of primary BrCa cell clusters (sh-CD44) embedded in 3D basement culture system, premixed with TAMs (Red channel), was recorded by time-lapse microscopy from 0h to 24h.

**Movie 5:** (Related to Fig.3G) Representative intravital images of subcutaneous xenografts derived from MCF7/sh-Control. Tomato lectin (DyLight649, Cat. L32472, Thermo Fisher) was injected via tail vein to label vascular structures.

**Movie 6:** (Related to Fig.3G) Representative intravital images of subcutaneous xenografts derived from MCF7/sh-Control, which was injected with THP1-derived M2-like macrophages, showing cohesive-invading MCF7/GFP^+^ cells approaching around vascular vessel. Tomato lectin (DyLight649, Cat. L32472, Thermo Fisher) was injected via tail vein to label vascular structures.

**Movie 7:** (Related to Fig.3G) Representative intravital images of subcutaneous xenografts derived from MCF7/sh-CD44 cells in vivo. Tomato lectin (DyLight649, Cat. L32472, Thermo Fisher) was injected via tail vein to label vascular structures.

**Movie 8:** (Related to Fig.3G) Representative intravital images of subcutaneous xenografts derived from MCF7/sh-CD44 cells in vivo, which was injected with THP1-derived M2-like macrophages. Tomato lectin (DyLight649, Cat. L32472, Thermo Fisher) was injected via tail vein to label vascular structures.

**Movie 9:** (Related to Fig.3H) Representative multi-photon confocal images of thick tissues from subcutaneous xenografts (MCF7/sh-Control). Tumor tissues cut for 10- to 12-µm paraffin sections, were examined using fluorescence microscopy. Tissue labeled by CD31 (Red) was examined by multi-photon confocal imaging.

**Movie 10:** (Related to Fig.3H) Representative multi-photon confocal images of thick tissues from subcutaneous xenografts (MCF7/sh-Control + THP1-derived M2-like macrophages). Tumor tissues cut for 10- to 12-µm paraffin sections, were examined using fluorescence microscopy. Tissue labeled by CD31 (Red) was examined by multi-photon confocal imaging.

**Movie 11:** (Related to Fig.3H) Representative multi-photon confocal images of thick tissues from subcutaneous xenografts (MCF7/sh-CD44). Tumor tissues cut for 10- to 12-µm paraffin sections, were examined using fluorescence microscopy. Tissue labeled by CD31 (Red) was examined by multi-photon confocal imaging.

**Movie 12:** (Related to Fig.3H) Representative multi-photon confocal images of thick tissues from subcutaneous xenografts (MCF7/sh-CD44 + THP1-derived M2-like macrophages). Tumor tissues cut for 10- to 12-µm paraffin sections, were examined using fluorescence microscopy. Tissue labeled by CD31 (Red) was examined by multi-photon confocal imaging.

**Supplemental Table 1 Correlation between CD206, CD44, CD31 expression and the clinic-pathological characteristics in the studied cohort**

| Tissue  marker |  | CD206 expression (IOD) | | Significance | CD44 expression (IOD) | | Significance | CD31 expression (IOD) | | Significance |
| --- | --- | --- | --- | --- | --- | --- | --- | --- | --- | --- |
|  | n | Median | 95% CI | *P* value | Median | 95% CI | *P* value | Median | 95% CI | *P* value |
| Age |  |  |  |  |  |  |  |  |  |  |
| ＜50y | 31 | 7889 | 7434-13788 | 0.2514 | 2321 | 2790-6397 | 0.4436 | 1817 | 2147-3499 | 0.0814 |
| ≥50y | 54 | 6960 | 6982-10453 |  | 4746 | 4066-6878 |  | 2992 | 3049-4415 |  |
| Tumor size |  |  |  |  |  |  |  |  |  |  |
| ＜3cm | 54 | 8721 | 7994-12197 | 0.2534 | 3499 | 3558-6133 | 0.4646 | 2267 | 2563-3908 | 0.3904 |
| ≥3cm | 31 | 5720 | 5840-10579 |  | 3913 | 2618-7752 |  | 3750 | 2939-4435 |  |
| Histologic grade |  |  |  |  |  |  |  |  |  |  |
| Ⅰ | 3 | 11588 | -4637-29849 | 0.0676 | 1404 | -6564-12597 | 0.3918 | 1241 | 595.1-1731 |  |
| Ⅱ | 43 | 6246 | 5623-9605 |  | 2366 | 2840-5950 |  | 2026 | 2095-3162 |  |
| Ⅱ-Ⅲ | 29 | 8761 | 7712-12799 |  | 5305 | 4163-8020 |  | 4314 | 3872-5809 |  |
| Ⅲ | 10 | 15070 | 6225-21180 |  | 4868 | 2575-10065 |  | 2215 | 1682-4748 |  |
| Lymph node metastasis |  |  |  |  |  |  |  |  |  |  |
| Yes | 14 | 7086 | 4735-14744 | 0.8535 | 5374 | 2560-8163 | 0.8661 | 3674 | 3015-5824 | 0.0714 |
| No | 71 | 7120 | 7672-11012 |  | 3575 | 3897-6324 |  | 2353 | 2666-3733 |  |
| Tumor with microemboli |  |  |  |  |  |  |  |  |  |  |
| Yes | 42 | 8519 | 8689-13398 | 0.0420* | 5497 | 5235-8844 | 0.0005* | 4848 | 4008-5281 | ＜0.0001* |
| No | 43 | 5466 | 5768-9881 |  | 2206 | 2272-4344 |  | 1638 | 1602-2769 |  |

*Indicated statistical significance (*p* <0.05)

**Supplemental Table 2 List of antibodies used in the study**

| **Protein** | **Catalog No.** | **Clone(if monoclonal)** | **Manufacturer** | **Application** | **Dilution** |
| --- | --- | --- | --- | --- | --- |
| EpCAM-PE-Cyanine7 | 25-5791-80 | G8.8 | Thermo Fisher | FACS | 0.2µg/test |
| CD45-FITC | 11-0451-82 | 30-F11 | Thermo Fisher | FACS | 0.5µg/test |
| F4/80-APC | 17-4801-82 | BM8 | Thermo Fisher | FACS | 2µg/test |
| CD206-PE | 12-2061-82 | MR6F3 | Thermo Fisher | FACS | 0.2µg/test |
| CD44-eFluor 780 | 47-0441-82 | IM7 | Thermo Fisher | FACS | 0.25µg/test |
| CD44-APC | 17-0441-82 | IM7 | Thermo Fisher | FACS | 0.2µg/test |
| CD206 | ab64693 | N/A | Abcam | Immunohistochemistry | 1:5000 |
| CD44 | 103004 | N/A | Biolegend | Immunohistochemistry | 1:200 |
| CCL8 | 933203 | A15150G | Biolegend | Neutralization | 40μg/test  0.2µg/ml |
| CD44 | ab189524 | N/A | Abcam | Western blot | 1:1000 |
| CD31 | ab56299 | N/A | Abcam | Immunohistochemistry | 1:200 |
| CK | BM0030 | N/A | Boster | Immunohistochemistry | 1:400 |
| Ezrin | 3145 | N/A | cell signaling technology | Western blot | 1:1000 |
| pEzrin | 3726 | N/A | cell signaling technology | Western blot | 1:1000 |
| HAS1 | ab198846 | N/A | Abcam | Western blot | 1:1000 |
| Phospho-p38 MAPK (Thr180/Tyr182) | 4511 | N/A | cell signaling technology | Western blot | 1:1000 |
| p-MDM2(Ser166) | 3521 | N/A | cell signaling technology | Western blot | 1:1000 |
| β-catenin | ab32572 | N/A | Abcam | Immunofluorescence | 1:200 |
| p-Myosin | 3671 | N/A | cell signaling technology | Immunofluorescence | 1:200 |
| p-ERM | 3726 | N/A | cell signaling technology | Immunofluorescence | 1:200 |
| GAPDH | Mab-5465-100 | N/A | Multi Sciences | Western blot | 1:1000 |

**Supplemental Table 3 List of primers used in the study**

| Primer |  | Sequence: 5’-3’ |
| --- | --- | --- |
| CCR4 | Forward | GGAAGGTATCAAGGCATTTGGG |
|  | Reverse | GTACACGTCCGTCATGGACTT |
| CCL8 | Forward | TCTACGCAGTGCTTCTTTGCC |
|  | Reverse | AAGGGGGATCTTCAGCTTTAGTA |
| TNFα | Forward | ATGTCTCAGCCTCTTCTCATTC |
|  | Reverse | GCTTGTCACTCGAATTTTGAGA |
| IL-6 | Forward | CTCCCAACAGACCTGTCTATAC |
|  | Reverse | CCATTGCACAACTCTTTTCTCA |
| IL-1Rα | Forward | GGTGCCTATTGACCTTCATAGT |
|  | Reverse | GATATCATCTCCAGACTTGGCA |
| IL-10 | Forward | GCTCTTACTGACTGGCATGAG |
|  | Reverse | CGCAGCTCTAGGAGCATGTG |
| CXCL12 | Forward | TGCATCAGTGACGGTAAACCA |
|  | Reverse | TTCTTCAGCCGTGCAACAATC |
| CCL22 | Forward | AGGTCCCTATGGTGCCAATGT |
|  | Reverse | CGGCAGGATTTTGAGGTCCA |
| CCL2 | Forward | TTAAAAACCTGGATCGGAACCAA |
|  | Reverse | GCATTAGCTTCAGATTTACGGGT |
| EGF | Forward | AGCATCTCTCGGATTGACCCA |
|  | Reverse | CCTGTCCCGTTAAGGAAAACTCT |
| GAPDH | Forward | AGGTCGGTGTGAACGGATTTG |
|  | Reverse | TGTAGACCATGTAGTTGAGGTCA |
| CD44 | Forward | AGTCACAGACCTGCCCAATG |
|  | Reverse | GCAGGGATTCTGTCTGTGCT |
| c-Myc | Forward | CCTCCACTCGGAAGGACTATC |
|  | Reverse | TGTTCGCCTCTTGACA TTCTC |
| Zyx | Forward | CAGCAGTTCTACAGTCTGGAG |
| Zyx | Reverse | GTACTGCTTGTGGTAGTCGG |
| PLAU | Forward | AATTTCAGTGTGGCCAAAAGAC |
|  | Reverse | GTCCTCCTTCTTTGGGTAATCA |
| Msn | Forward | GATGCTGTCCTGGAATATCTGA |
|  | Reverse | TCTGCTCATAGATGTTGAGACC |
| ADAM8 | Forward | TGAATCACGTGGACAAGCTATA |
|  | Reverse | GAACCTGTCCTGACTATTCCAA |
| PLAUR | Forward | CTATCGGACTGGCTTGAAGATC |
|  | Reverse | CAGGAAATGCATTCGAGGTAAC |
| HDAC1 | Forward | ATCCGCATGACTCATAATTTGC |
|  | Reverse | GGATGGAGCGCAAGAATTTAAT |
| JUNB | Forward | CTTCTACCACGACGACTCATAC |
|  | Reverse | TTTCAGGAGTTTGTAGTCGTGT |
| Axl | Forward | AGATTTATGACTATCTGCGCCA |
|  | Reverse | TGACATAGAGGATTTCGTCAGG |
| EPCAM | Forward | GTCTGTGAAAACTACAAGCTGG |
|  | Reverse | CAGTATTTTGTGCACCAACTGA |
| CDH2 | Forward | CGATAAGGATCAACCCCATACA |
|  | Reverse | TTCAAAGTCGATTGGTTTGACC |
