# Supplementary material for: Activation of CD44 signaling in leader cells induced by tumor-associated macrophages drives collective detachment in luminal breast carcinomas
Source: Cell Death Dis. 2022 Jun 9;13(6):540. doi: 10.1038/s41419-022-04986-4 (PMC9184589; doi:10.1038/s41419-022-04986-4)
Supplement: Supplementary file 3 — Supplementay data 3 [file 41419_2022_4986_MOESM3_ESM.pdf]

Raw figure of Fig.3A

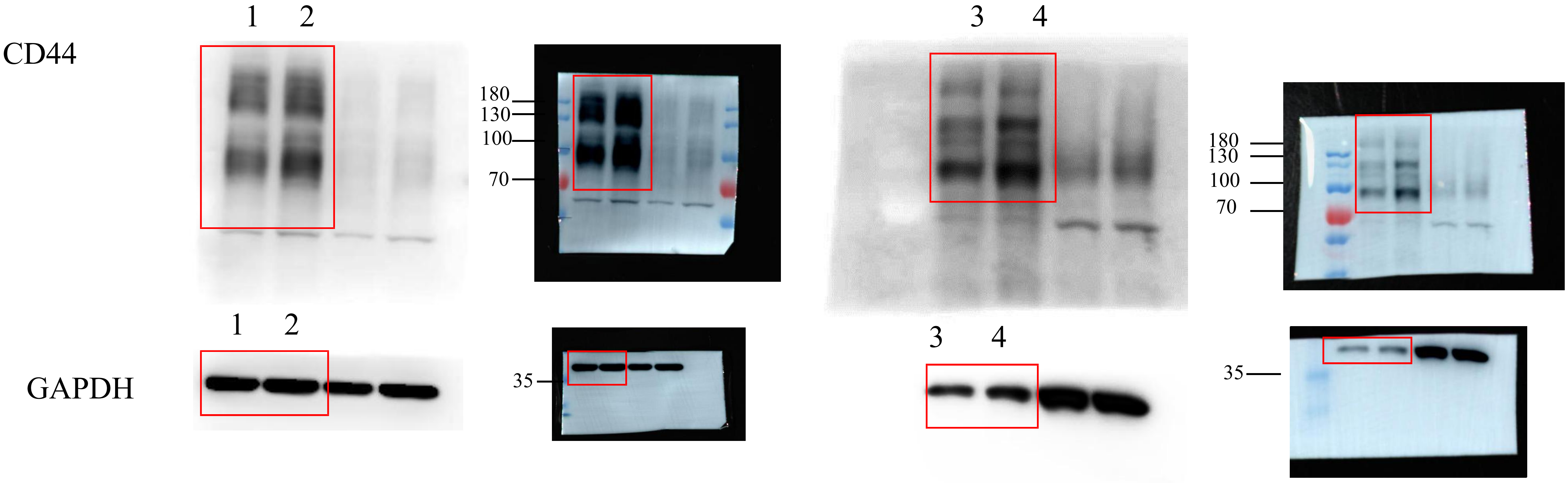

1 MCF7
2 MCF7 THP-1-derived M2-like MΦ

3 T47D
4 T47D THP-1-derived M2-like MΦ

Raw figure of Fig.3C

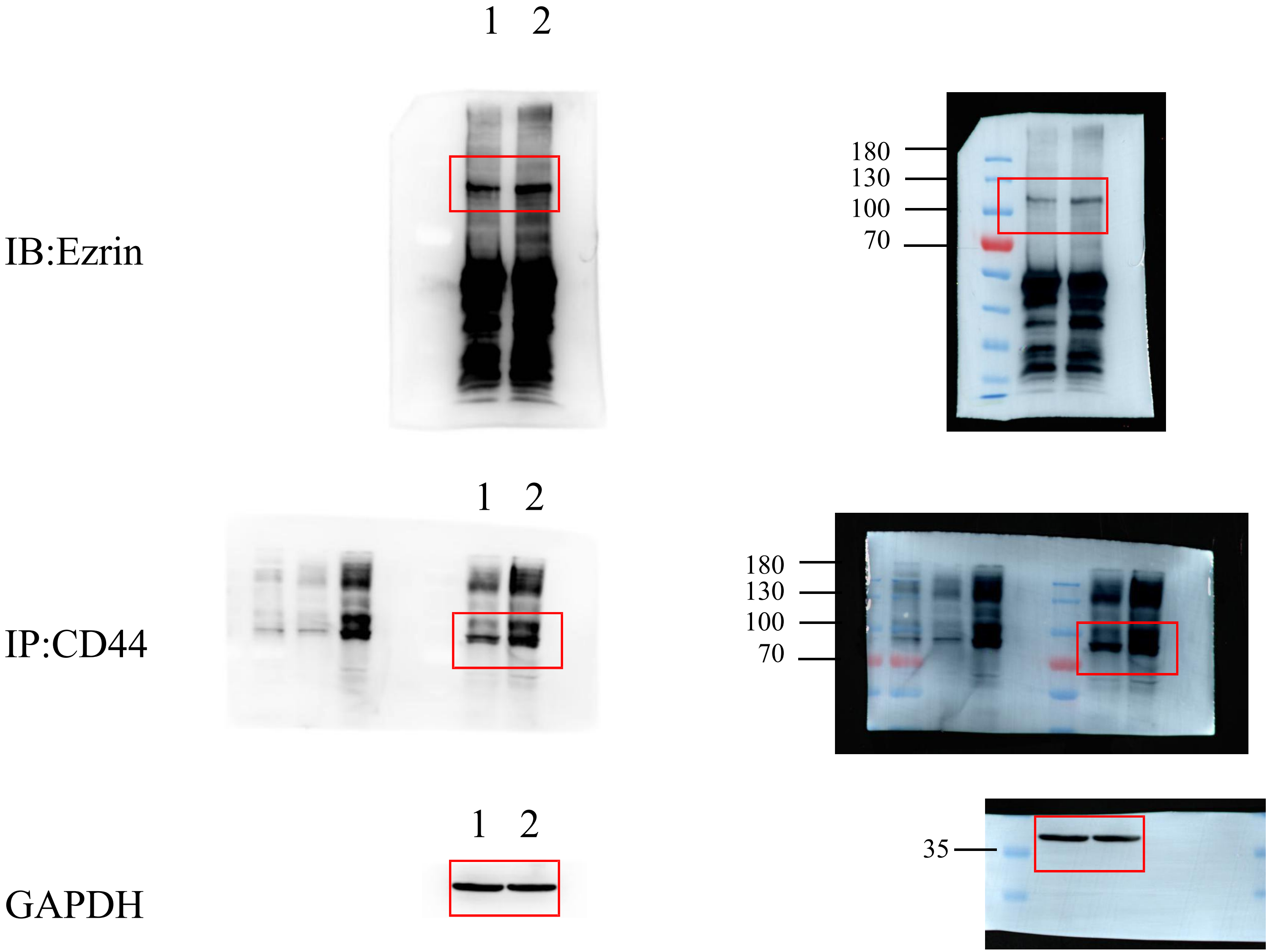

1 MCF7
2 MCF7 THP-1-derived M2-like MΦ

Raw figure of Fig.5A

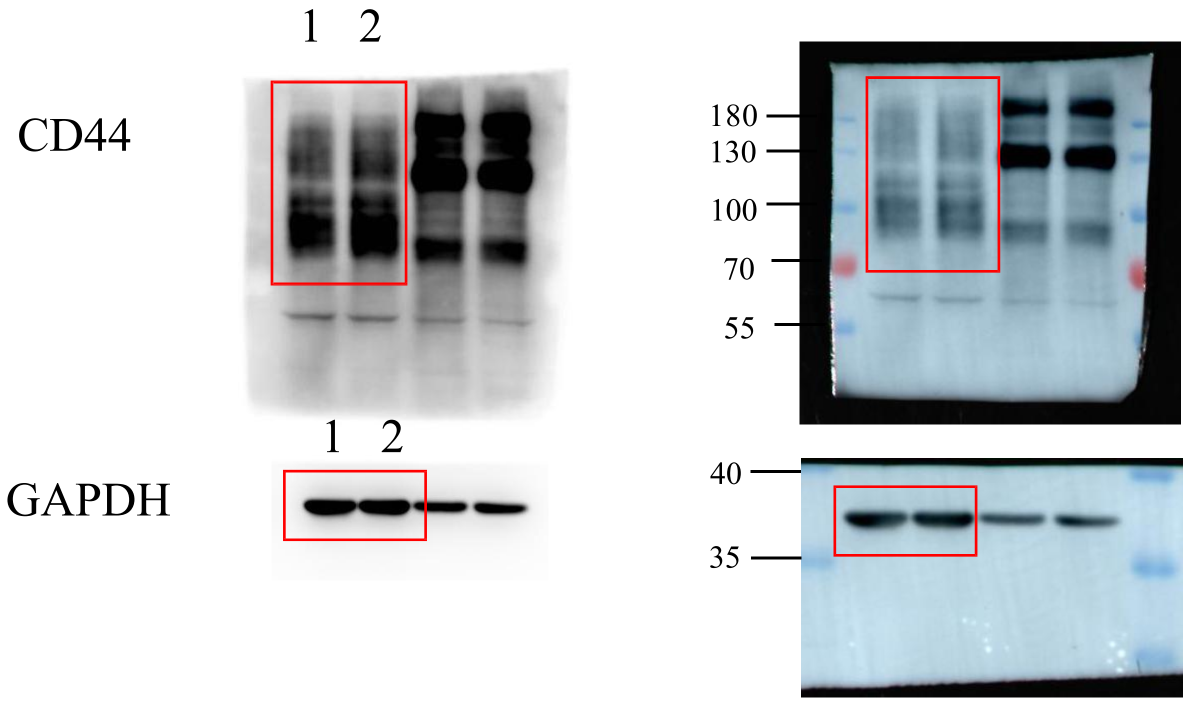

1 MCF7 control
2 MCF7 CCL8

Raw figure of Fig.5B

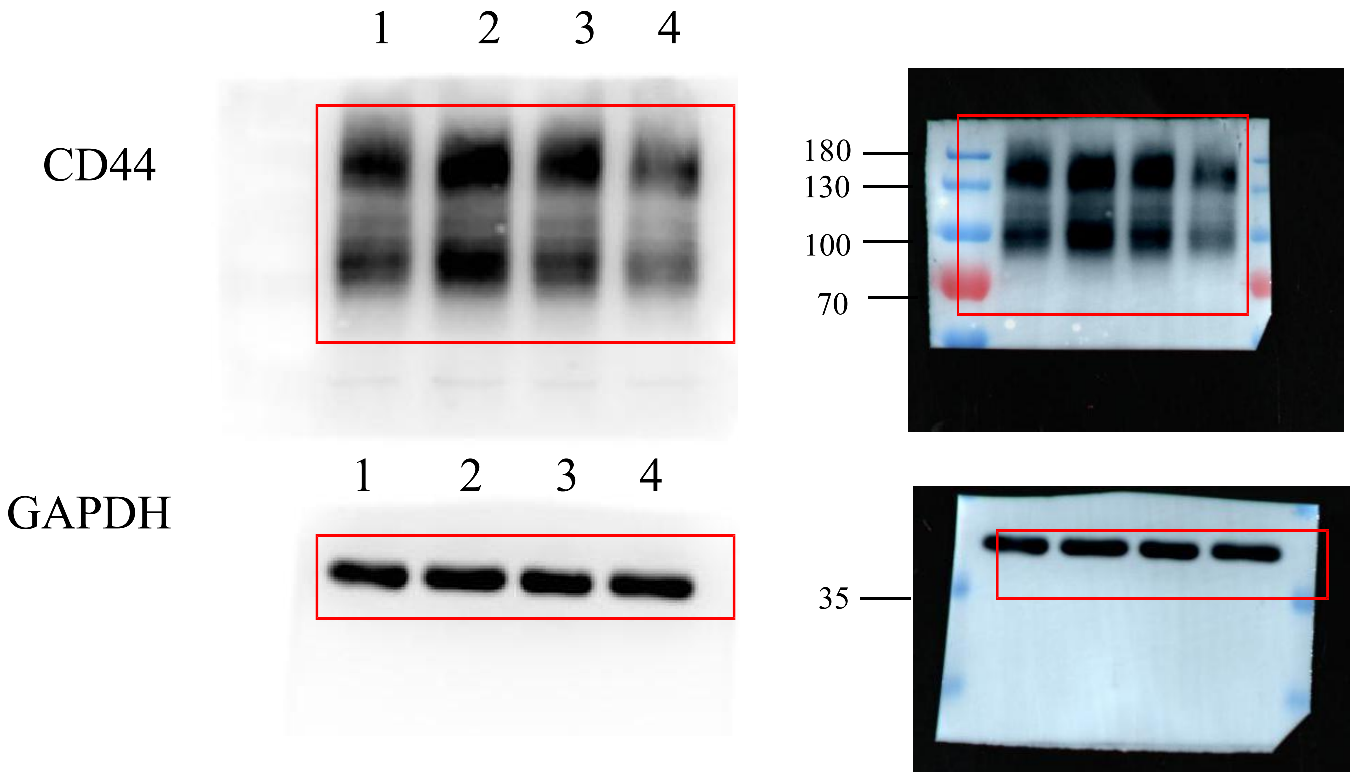

1 MCF7 control
2 MCF7 THP-1-derived M2-like MΦ control
3 MCF7 THP-1-derived M2-like MΦ NlgG
4 MCF7 THP-1-derived M2-like MΦ CCL8 Ab

Raw figure of Fig.5E

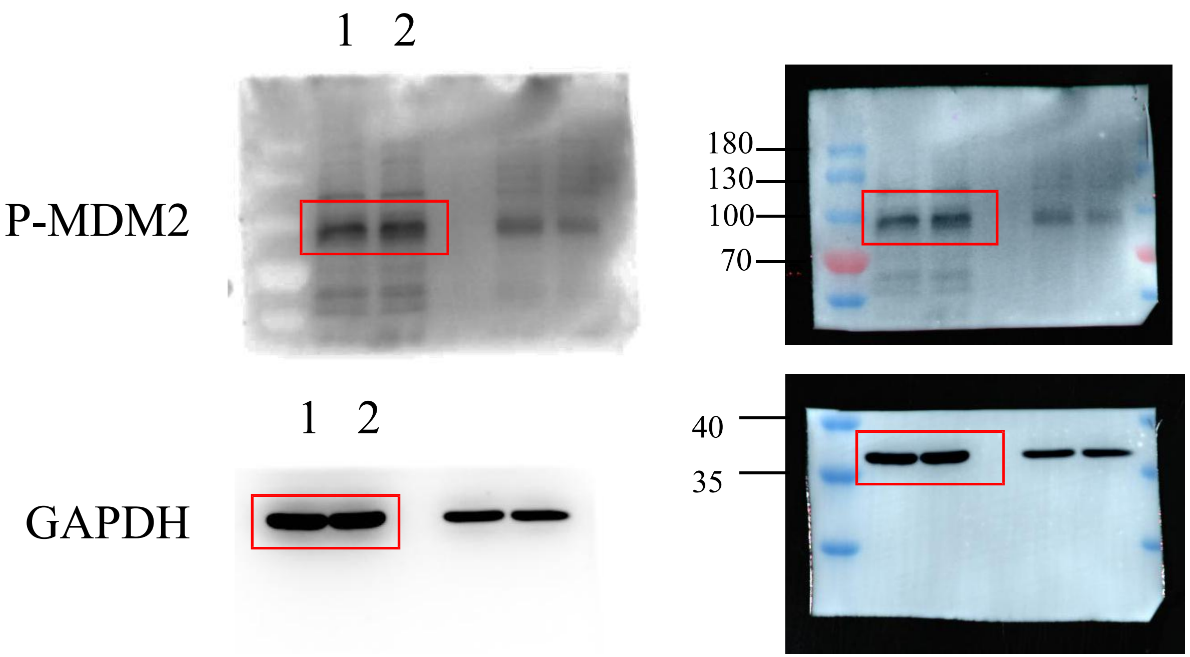

1 MCF7 control
2 MCF7 CCL8

Raw figure of Fig.5F

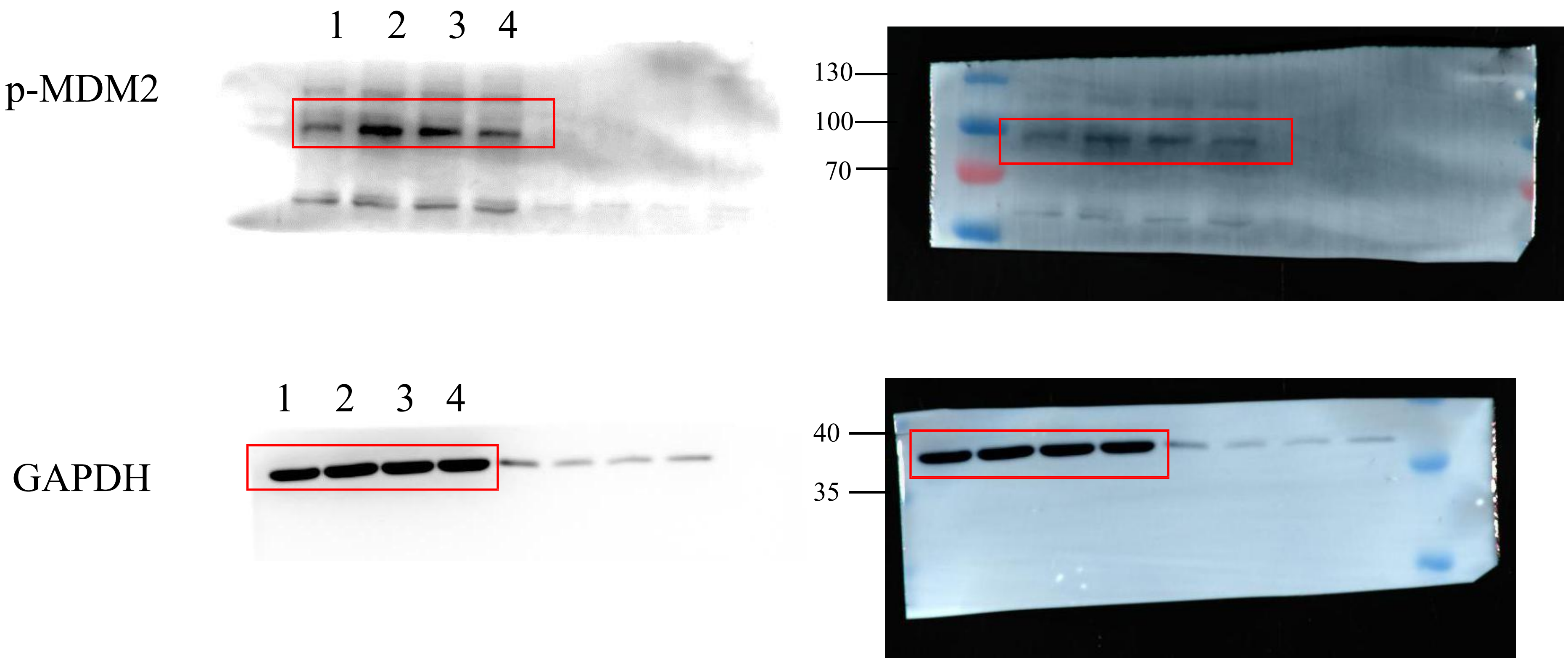

1 MCF7 control
2 MCF7 THP-1-derived M2-like MΦ control
3 MCF7 THP-1-derived M2-like MΦ NlgG
4 MCF7 THP-1-derived M2-like MΦ CCL8 Ab

Raw figure of Fig.5G

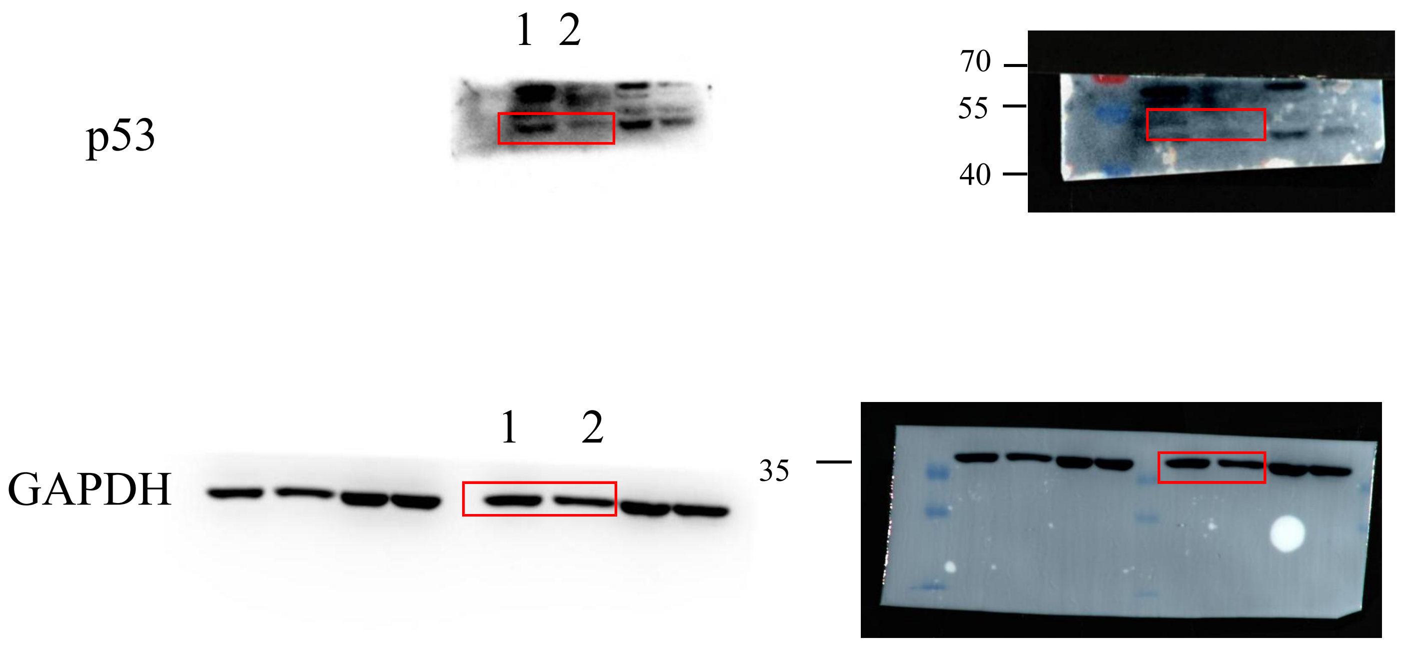

1 MCF7 Control
2 MCF7 THP-1-derived M2-like MΦ

Raw figure of Fig.5H

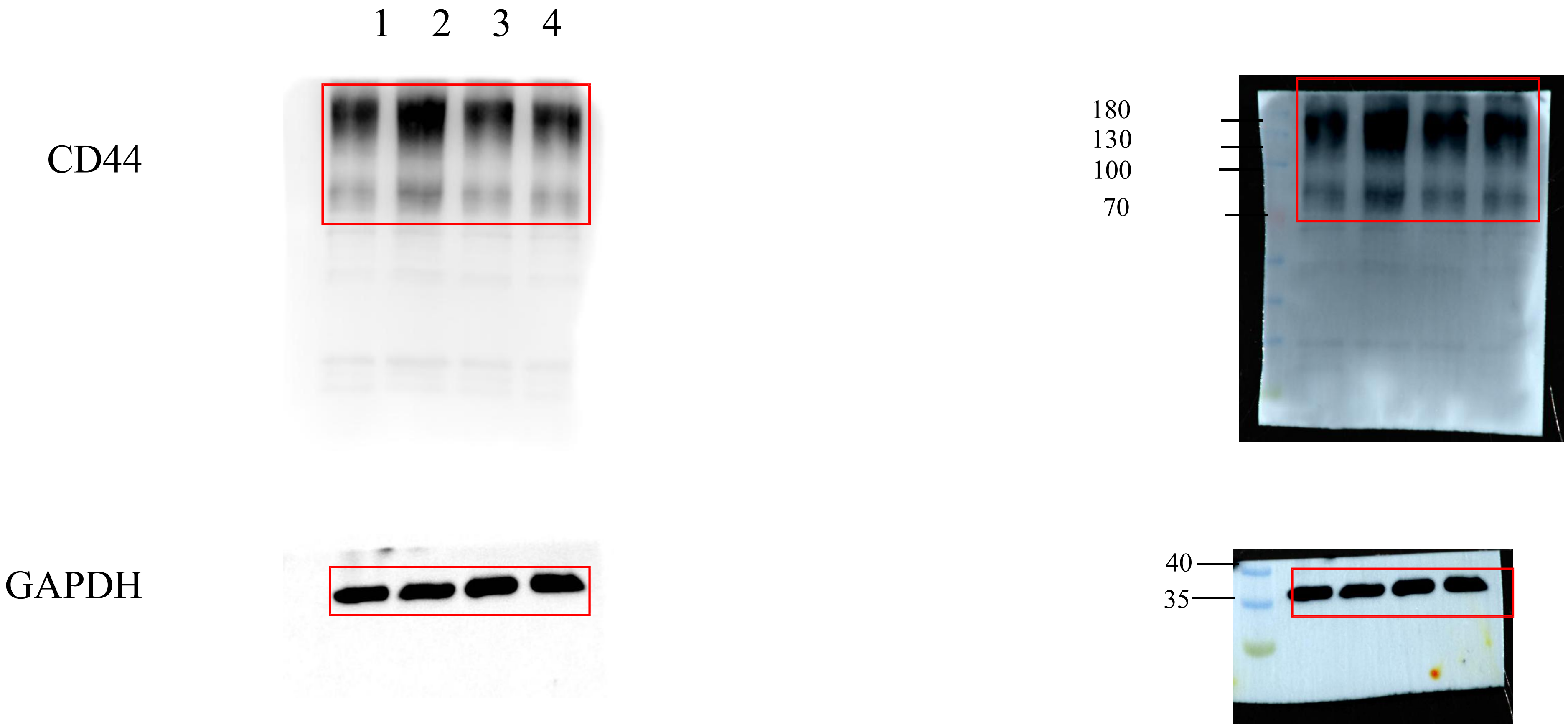

1 Primary BrCa cells si-Control
2 Primary BrCa cells si-Control CCL8
3 Primary BrCa cells si-MDM2
4 Primary BrCa cells si-MDM2 CCL8

Raw figure of Fig.6A

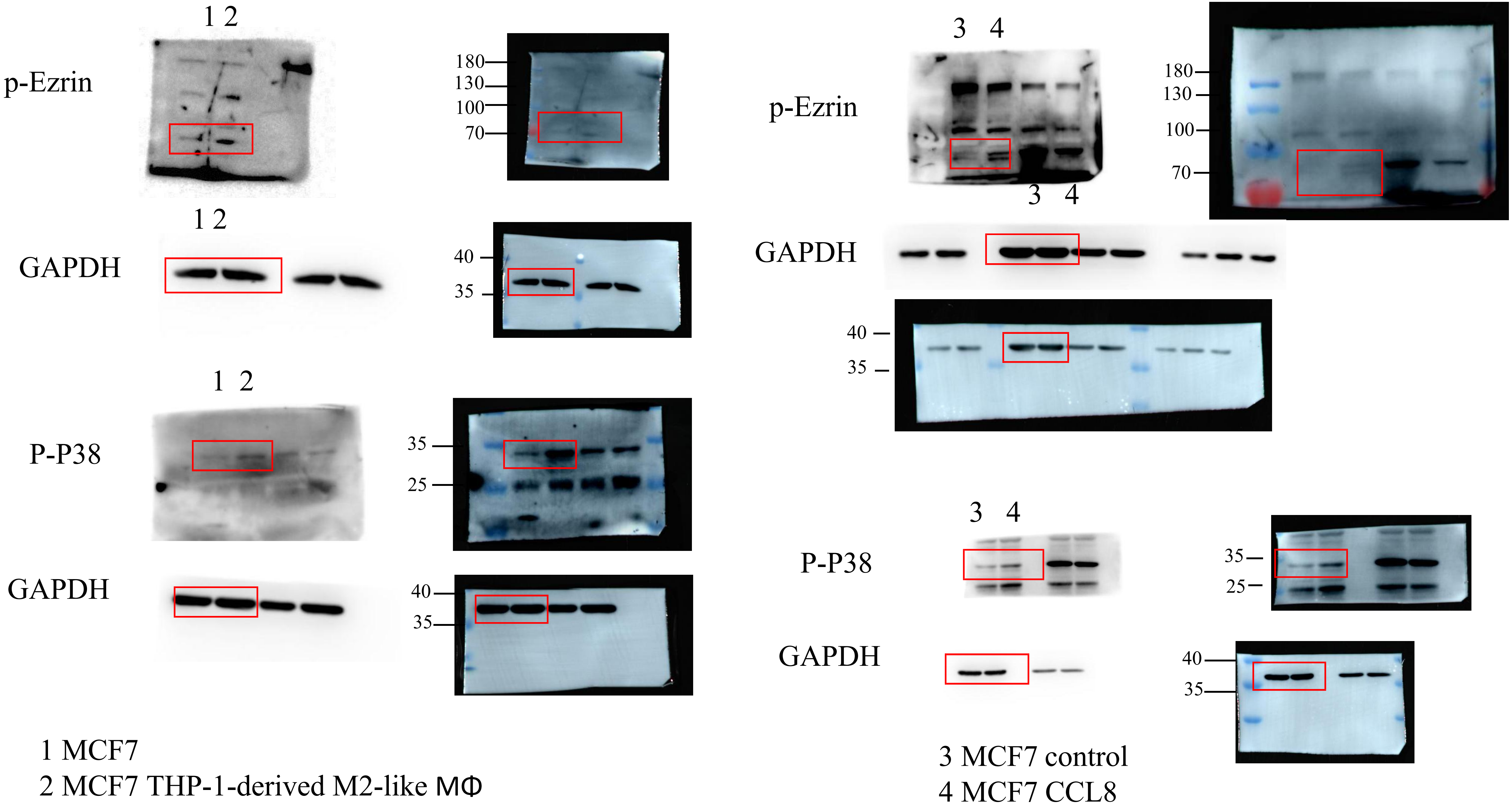

Raw figure of Fig.6B

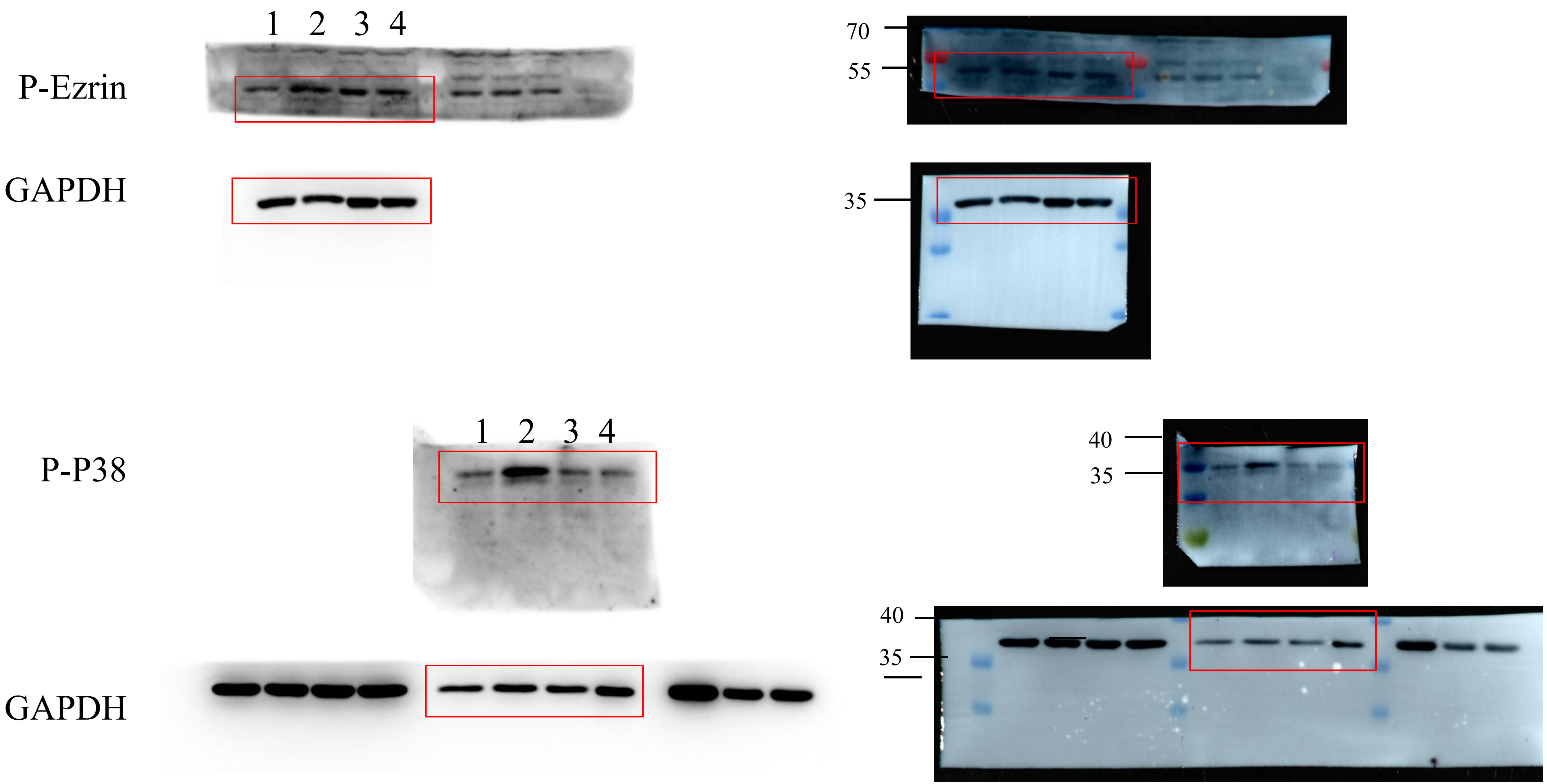

1 MCF7 sh-control
2 MCF7 sh-control THP-1-derived M2-like MΦ
3 MCF7 sh-CD44
4 MCF7 sh-CD44 THP-1-derived M2-like MΦ

Raw figure of Fig.6E

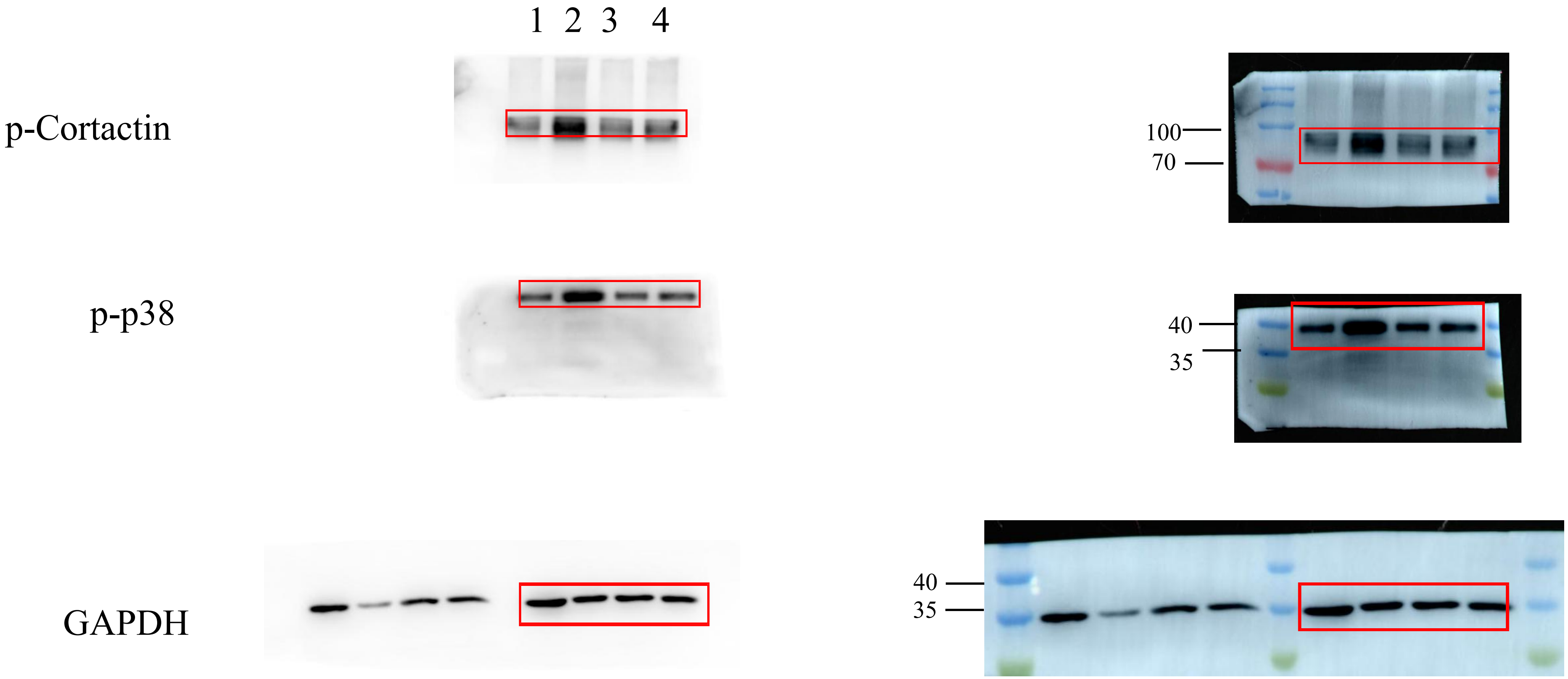

1 Primary BrCa cells si-Control
2 Primary BrCa cells si-Control CCL8
3 Primary BrCa cells si-Ezrin
4 Primary BrCa cells si-Ezrin CCL8

Raw figure of Fig.6F

p-Cortactin

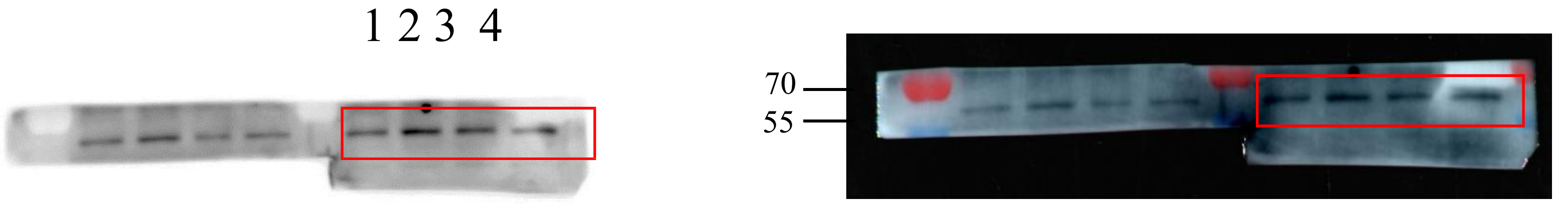

GAPDH

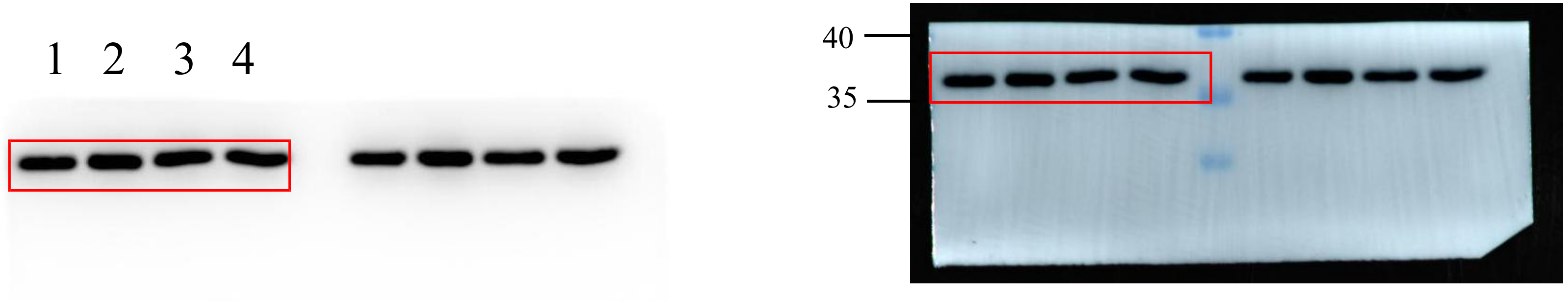

1 Primary BrCa cells si-Control
2 Primary BrCa cells si-Control CCL8
3 Primary BrCa cells si-p38
4 Primary BrCa cells si-p38 CCL8

Raw figure of Fig.S3

IB:Merlin

1 2

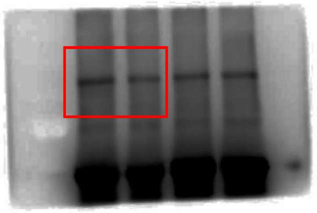

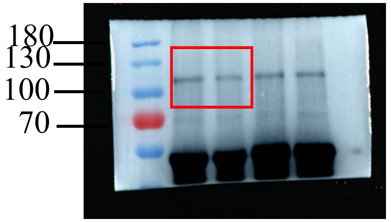

1 2

GAPDH

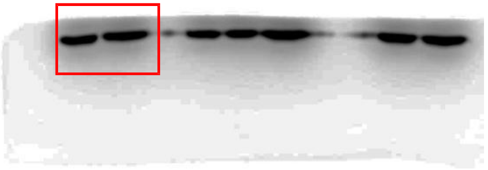

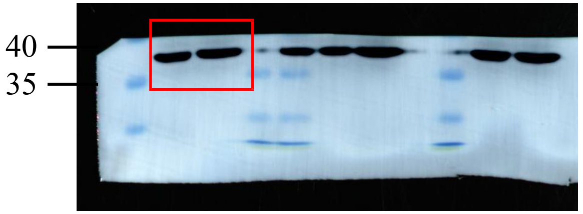

1 MCF7

2 MCF7 THP-1-derived M2-like MΦ
